# Cationic Polymers as Transfection Reagents for Nucleic Acid Delivery

**DOI:** 10.3390/pharmaceutics15051502

**Published:** 2023-05-15

**Authors:** Xiaomeng Cai, Rui Dou, Chen Guo, Jiaruo Tang, Xiajuan Li, Jun Chen, Jiayu Zhang

**Affiliations:** 1 CAS Key Laboratory for Biomedical Effects of Nanomaterials and Nanosafety, Multi-Disciplinary Research Division, Institute of High Energy Physics and University of Chinese Academy of Sciences (UCAS), Chinese Academy of Sciences (CAS), Beijing 100049, China; 2 Beijing Institute of Genomics, Chinese Academy of Sciences (CAS), China National Center for Bioinformation, Beijing 100101, China

**Keywords:** cationic polymers, DNA, mRNA, siRNA, delivery

## Abstract

Nucleic acid therapy can achieve lasting and even curative effects through gene augmentation, gene suppression, and genome editing. However, it is difficult for naked nucleic acid molecules to enter cells. As a result, the key to nucleic acid therapy is the introduction of nucleic acid molecules into cells. Cationic polymers are non-viral nucleic acid delivery systems with positively charged groups on their molecules that concentrate nucleic acid molecules to form nanoparticles, which help nucleic acids cross barriers to express proteins in cells or inhibit target gene expression. Cationic polymers are easy to synthesize, modify, and structurally control, making them a promising class of nucleic acid delivery systems. In this manuscript, we describe several representative cationic polymers, especially biodegradable cationic polymers, and provide an outlook on cationic polymers as nucleic acid delivery vehicles.

## 1. Introduction

Transfection is the process of delivering exogenous nucleic acids into cells [1]. Its purpose is to enable the protein encoded by the foreign gene to be expressed in the cell. These coding sequences are often carried into cells by plasmid DNA, in order to study their unknown function or for specific therapeutic purposes. In addition, siRNAs that reduce gene expression are also targets for nucleic acid transfection [2]. Through the knockdown effect of siRNA, researchers can manipulate the expression of healing genes to study gene function and interactions. siRNA plays an important role in cancer research, gene therapy, tissue engineering, etc. [3,4,5]. mRNA was once considered not ideal for gene therapy drugs, due to its ease of degradation. However, researchers have improved its stability through chemical modification, making it an ideal nucleic acid drug for expressing foreign genes [6,7]. mRNA vaccines have been used in the prevention of COVID-19, and many mRNA drugs for cancer treatment are in development.

The efficiency of naked nucleic acid molecules to be uptaken by cells is extremely low. This is because nucleic acids are hydrophilic, negatively charged biomolecules that have difficulties in approaching hydrophobic, negatively charged lipid cell membranes [8]. As a result, nucleic acid molecules must be delivered into cells via a vector. There are two kinds of nucleic acid vectors: viral and non-viral vectors. In terms of transfection effectiveness and packaging capacity, viral vectors perform well [9]. However, the disadvantages of viral vectors cannot be ignored, such as the tendency to trigger inflammatory responses and gene mutations [10]. Non-viral vectors, on the other hand, have an abundant source of materials, a controlled chemical structure, and can be easily prepared in large quantities; as a result, they have an irreplaceable role in nucleic acid transfection [11].

**Figure 1:**
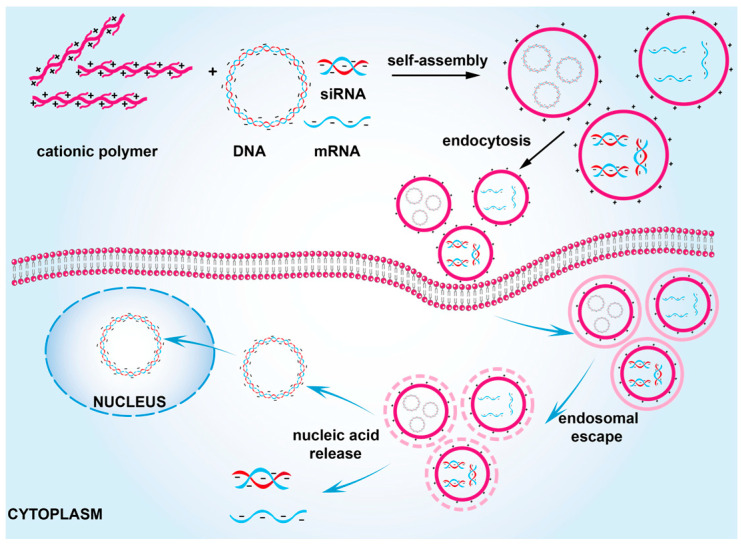
Cationic polymers are used as nucleic acid delivery vehicles.

Cationic polymers are a type of non-viral vector (Figure 1). A common feature of their structures is the presence of many positively charged groups in the molecule, which are protonated into positively charged polymers. Cationic polymers can bind nucleic acids through electrostatic interactions and condense them into small nanoparticles [12]. Positive charges improve interactions with negatively charged cell membranes, and aid polyplexes in escaping endosomes before lysosomal degradation occurs. [13]. Subsequently, polyplexes escape from the endosome by the proton sponge effect that is mediated by positively charged groups on the cationic polymer [14]. Furthermore, nucleic acids must dissociate from cationic polymers before finally performing their function [15]. 

Successful nucleic acid transfection requires high transfection efficiency and low cytotoxicity. Both characteristics must be considered when determining whether cationic polymers are suitable nucleic acid transfection reagents. It is generally accepted that the higher the molecular weight of the cationic polymer, the greater its ability to encapsulate nucleic acids and be uptaken by cells, and the poorer the cell viability and nucleic acid release. On the other hand, polymers with lower molecular weights have a reduced ability to condense nucleic acids and be uptaken by cells, but are better in terms of cytotoxicity and nucleic acid release. Therefore, the molecular weight of the cationic polymer should be carefully considered during transfection. Some chemical modifications, such as PEGylation and cholesterol modification, significantly improve the properties of polymers [16]. Furthermore, biodegradable materials are an effective means of reducing cytotoxicity [17]. In this review, we present several cationic polymers (Table S1). Moreover, we briefly describe the prospects of cationic polymers as delivery vehicles in the field of gene therapy.

## 2. Cationic Polymers

### 2.1. Synthetic Polymers

Synthetic polymers are a class of polymers produced artificially by the chemical polymerization reaction of small molecules or modification of natural polymers, as distinct from naturally occurring polymers. Depending on the reaction monomer and the reaction mechanism, different kinds of synthetic polymers can be obtained. In addition to some of the most common conventional cationic polymers, there are some special cationic polymers and some new types of cationic polymers. Here is an overview of synthetic cationic polymers.

#### 2.1.1. Conventional Polymers

- DEAE-dextran

DEAE-dextran is a chemically modified analog of dextran. By modification with diethylaminoethylen groups, the amidation of the dextran chains is readily protonated, which allows it to self-assemble into nanoparticles with negatively charged nucleic acids. DEAE-dextran was the first cationic polymer to be used for nucleic acid transfection. Early in the 1950s, it greatly enhanced the transfection of poliovirus and SV40 viral DNA in mammalian cells [18,19]. Subsequently, DEAE-dextran was widely used in the transfection of RNAs or DNAs [20,21]. However, DEAE-dextran did not perform as an optimal candidate for the following reasons: 1. The transfection efficiency was much lower than that of other agents such as liposomes [22,23]; 2. The cytotoxicity and immunogenicity of DEAE-dextran could not be ignored [24,25,26]. Therefore, more new candidates needed to be discovered.

- Poly-amino acids

PLL (poly-L-Lysine) is a positively charged poly-amino acid under physiological conditions. It can bind to plasmid DNAs and condense them into compact particles when the chain length is more than 20 residues [27]. Researchers have found that the covalent attachment of ligands on PLL significantly enhances endocytosis via receptor-mediated pathways. For example, asialoorosomucoid (ASOR) is a ligand for the sialoglycoprotein receptor that is expressed on hepatocytes. When ASOR was covalently attached to PLL, the receptor-mediated endocytosis and the cellular uptake of plasmids significantly increased [28]. Furthermore, PLL linked with folic acid or transferrin has been developed, and has made substantial progress in the transfection of pDNAs into cancer cells [29,30]. Another important poly-amino acid is PLO (poly-L-ornithine). PLO shares the property of PLL, but achieves a 10-fold increase in transfection efficiency compared to PLL [31]. Ramsay and Gumbleton demonstrated that PLO condensed pDNA at lower charge (+/−) ratios than PLL, and PLO/pDNA complexes were found to be more resistant to disruption than PLL/pDNA complexes at the same polycation/pDNA mass ratio [32]. However, due to their poor ability to mediate endosomal escape, the transfection efficiency of positively charged poly-amino acids remains low.

- PAMAM

**Figure 2:**
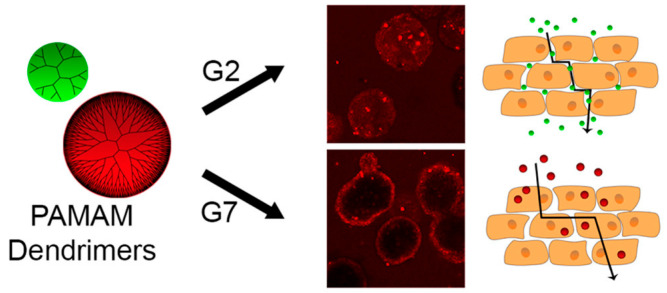
Size control and surface charge control of PAMAM dendrimers for per meation mechanism Reprinted with permission from Ref. [41]. Copyright 2016, American Chemical Society.

Poly(amidoamine) (PAMAM) dendrimers are repetitive units of three-dimensional branched macromolecules with plenty of active amine groups on the surface [33]. Their molecular size and surface charge can be well-defined (Figure 2). PAMAM dendritic polymers are synthesized with a divergent approach that involves the use of different initiators containing functional groups such as ethylenediamine (EDA), ammonium (NH_3_), and triethanolamine (TEA) [34]. The pKa of PAMAM is around 6.0, which is nearly the same as the endosome [35]. When the PAMAM/nucleic acids complex is endocytosed, the amino groups are protonated, resulting in a significant influx of chloride ions. The osmotic pressure of the endosome then rises, finally breaking the endosome. This procedure is called the proton sponge effect, which makes it easier for the complex to escape from the endosome, thus increasing the efficacy of nucleic acid transfection [14]. Both pDNA and siRNA can be encapsulated into condensed forms by PAMAM. The plasmids expressing luciferase and beta-galactosidase were transferred into mammalian cells with high efficiency [36,37]. siRNA condensed by PAMAM into nanoparticles were resistant to RNases, and effectively reduced mRNA and protein levels [38]. PAMAM dendrimers caused significant necrosis and mild apoptosis [39]. Surface modification with substances such as polysaccharides, PEG, acetate, and others can reduce cytotoxicity. Furthermore, the alternative architecture significantly enhanced the biocompatibility of PAMAM [40].

- PPI (poly-(propylenimine))

Polyacrylimide (PPI) dendrimers are the first family of dendrimers that were synthesized on an industrial kilogram scale. Typically, they were synthesized with ethylene diamine (EDA) and diaminobutane (DAB) as the core by a divergent approach [42]. As a gene-delivery agent, PPI has been widely explored. It is a highly branched spherical dendrimer with primary amino groups at the periphery, and is hydrophobic in the interior [43]. The generation of PPI is related to the cytotoxicity and the surface charge. Higher generation numbers represent greater nucleic acid-binding capacity and higher cytotoxicity [43]. Researchers have revealed that the G4 dendrimers were optimized with maximum efficiency and minimal toxicity when delivering siRNA [34]. Moreover, genes transfected by PPI are able to be expressed in the liver. Several modifications were used to reduce the cytotoxicity. For example, PPI was modified using a fluorination strategy to achieve efficient and low cytotoxic gene transfection. In that study, PPI was able to transfect cells at a very low N/P ratio, with a higher transfection efficiency than commercial reagents, and with a cell viability of over 90% [44].

- PEI (polyethylenimine)

PEI (polyethylenimine) was reported to perform with high transfection efficiency when forming a complex with pDNA. Its condensation ability and surface charge rose as the molecular weight of the compound grew [45]. Similar to PAMAM, PEI induces the proton sponge effect. Its buffering capacity causes osmotic swelling and endosome rupture, allowing the polyplexes to escape into the cytoplasm. Other factors, in addition to the proton sponge effect, are thought to cause endosome escape. PEI, for example, can cause endosome membrane destabilization by generating hydrophilic pores in the lipid bilayer, resulting in endosome disruption [46]. Furthermore, it has been shown that when PEI is protonated, the polymer chain elongates due to electrostatic repulsion, causing polymer swelling [47,48]. Two kinds of PEI, branched PEI (BPEI) and linear PEI (LPEI), are commercially accessible. LPEI exhibits better transfection efficiency than BPEI, especially in non-dividing cells [49]. It is possible that linear PEI’s higher efficiency results from its inherent kinetic instability in salty environments [50]. Moreover, LPEI’s better nuclei delivery may be attributed to its higher efficiency, while BPEI complex has to wait for nuclear membrane degradation before entering [51].

It is worth noting that PEI’s molecular weight has an impact on cytotoxicity and gene transfer activity. Since PEI is not degradable inside the cell, the higher the molecular weight, the more cytotoxic it is. Furthermore, PEI with a higher molecular weight forms more stable polymers, making it easier to transfect but more difficult for it to release nucleic acids intracellularly. The complex generated by PEI with reduced molecular weight, on the other hand, is more difficult to transfect; however, it is easier for it to release nucleic acid. As a result, determining which molecular weight of PEI is more favorable cannot be achieved arbitrarily. However, some improvements make PEI more advanced in applications. Conjugation of low-molecular-weight (LMW) PEI with a biodegradable backbone, such as polyglutamic acid derivative (PEG-b-PBLG), significantly reduces the cytotoxicity and maintains high transfection efficiency [52]. Various non-toxic branching PEI derivatives were created by modifying amines with ethyl acrylate, acetylation of primary amines, or introducing negatively charged propionic or succinic acid groups into the polymer structure. The resulting chemicals were extremely successful at employing siRNA to knockdown target genes [53,54].

With the increasing demand for nucleic acid drugs, the demand for low toxicity and degradable delivery materials is becoming increasingly important. Many studies have focused on designing degradable PEI derivatives using low-molecular-weight (LMW) PEI and degradable linkers [55]. Degradable PEI can be classified into esters, disulfides, imines, amino formates, amides, ketones, and others. Due to the degradability of the linker, the synthesized PEI exhibits reduced cytotoxicity and can achieve triggered release of nucleic acid substances. For example, disulfide bonds are a popular strategy for creating redox-sensitive polymer gene carriers, which are easily degraded in the cellular environment due to high concentrations of glutathione. Therefore, connecting low-molecular-weight PEI with disulfide bonds can not only reduce toxicity, but also achieve intracellular release of nucleic acid substances, preventing problems such as rapid secretion or premature release that may cause biological safety issues or insufficient transfection efficiency.

- PDMAEMA (polymethacrylic acid N,N-dimethylaminoethyl ester)

PDMAEMA (polymethacrylic acid N,N-dimethylaminoethyl ester) was synthesized through atom transfer radical polymerization (ATRP). It was a cationic hydrogel that contains only tertiary amines. In 1996, PDMAEMA was first reported to transfect the nucleic acid into COS-7 cells [56]. As an interesting and smart cationic polymer, PDMAEMA is responsive to both temperature and pH, making it a promising carrier for the controlled release of drugs. When heated above LCST (lower critical temperature solution), PDMAEMA converts from a hydrophilic to a hydrophobic structure. The temperature range of LCST is typically between 32 and 53 °C, and its preset value is strongly associated with the pH of the environment. PDMAEMA with a molecular weight of more than 300 kDa efficiently condenses DNA into compact nanoparticles, while those less than 300 kDa cannot [57]. Linear, highly branched, and star-shaped structures are three kinds of PDMAEMA. The star-shaped PDMAEMA, according to previous studies, shows better transfection efficiency than the other two. Early studies suggest that PDMAEMA releases nucleic acids by breaking through the endosome via the proton sponge effect. However, confocal laser fluorescence microscopy (CSLM) and electron microscopy (EM) did not observe escaped polymers or plasmids, indicating that endosome escape is an inefficient process [58,59]. One speculation suggests that PDMAEMA may achieve nucleic acid release by affecting endosomal membrane instability. Membrane-disrupting peptide conjugation, such as INF-7 peptide derived from the influenza virus, enhanced endosome escape and, therefore, increased the transfection efficiency of plasmid DNA [60].

#### 2.1.2. Polyesters

- Poly(β-amino ester)

Poly(β-amino ester) (PBAE) is a cationic polymer synthesized with acrylates and amines via Michael addition reactions [61]. It was initially produced in 1983 and used as a gene carrier until 2000 [62]. PBAE is biodegradable, biocompatible, and pH-responsive; therefore, it has become an attractive candidate as a nucleic acid delivery reagent [63,64,65,66,67,68,69,70]. The polymer forms backbones with ester bonds that are readily degraded by hydrolysis reactions under physiological conditions. When hydrolyzed, the polymer breaks into small molecules, such as bis(β-amino acids) and diols, which are considered to be harmless to mammalian cells [62]. The surface charge density of PBAE is lower than that of other cationic polymers, which means its binding strength with nucleic acid is lower. As a result, the amount of PBAE required for nucleic acid binding should be significantly higher than that required for PEI, PPI, and other cation polymers. Even so, transfection with PBAEs as vectors is more efficient, due to its reduced cytotoxicity. Endcaps containing secondary amines or other amine-containing groups have been demonstrated to boost the binding capacity of PBAE and the overall transfection efficiency. Furthermore, PBAE can be converted from hydrophobic to hydrophilic in response to pH variations [71]. As a result of their hydrophobic nature, nanoparticles are more stable and compact, and interact with the cell membrane more easily [72]. 

**Figure 3:**
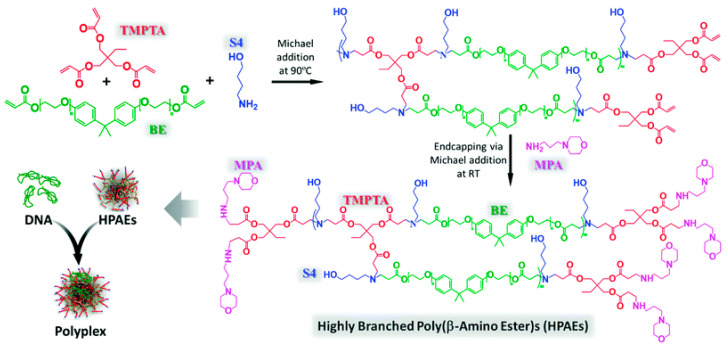
Synthesis of highly branched PBAE. Reprinted with permission from Ref. [73]. Copyright 2015, The Royal Society of Chemistry.

PBAEs can be divided into linear and branched structures [68]. Currently, thousands of linear PBAEs have been synthesized, most of which have demonstrated effective transfection efficiency. Triacrylates and an amino moiety with two reaction sites are used to make highly branched PBAEs (HPBAEs) (Figure 3) [68,73]. An advantage over LPBAEs is that the three-dimensional structure of HPBAEs protects DNA from enzymatic degradation [74]. In addition, both PBAEs can be further modified by including other chemicals via a predictable and simple synthesis procedure to produce varied PBAE copolymers with robust transfection and excellent safety. PBAEs are the most powerful alternative to viral vectors, due to their superior transfection performance. Therapeutic regimens that use PBAE as a vector are currently showing significant promise in the treatment and prevention of a variety of diseases. For one example, OM-PBAEs (oligopeptide end-modified PBAEs) transfected mRNA to antigen-present cells for immunotherapy against cancers and infectious illnesses [75]. As another example, end-modified (PBAE)-based nanoparticles transferred siRNA to human mesenchymal stem cells (HMSC) for osteogenic differentiation enhancement [76]. Furthermore, highly branched PBAEs delivered minicircle DNA for neurological disease treatment [77].

- PHP (Poly(4-hydroxy-L-proline ester))

PHP, which is made of hydroxyproline from natural sources such as collagen, gelatin, and other proteins, was the first polyester used as a gene carrier [78,79]. In a physiological setting, PHP can lose 50% of its initial molecular weight in less than two hours. However, the full breakdown of PHP into its equivalent monomer takes three months, with the breakdown product being a monomeric hydroxyproline [79]. Although PHP ester is quickly degraded when present alone in solution, it is more stable when complexed with DNA. PHP ester/pSV-gal complexes were transfected into CPAE cells to determine the viability of PHP ester as a gene delivery carrier. Since poly-L-lysine is the most popular polymer for gene transport, PHP ester’s transfection effectiveness was on par with that of poly-L-lysine. The effectiveness of transfection increased as the PHP ester concentration was raised above that of the DNA. PHP ester’s ability to transfect cells was unaffected by the presence of FBS. As a result of these findings, PHP ester was demonstrated to be a potential gene carrier.

- PAGA (poly[α-(4-aminobutyl)-l-glycolic acid])

PAGA is a useful biodegradable poly-cation. It degrades rapidly in aqueous solution, and the end product of the degradation is a monomer (l-oxylysine). It can strongly form complexes with DNA at charge ratios below 2 [80]. In the presence of 5% (*w*/*v*) glucose, PAGA was employed to assemble a complex with plasmid DNA expressing mouse interleukin-12 (pmIL-12). Forty-eight hours after intra-tumoral injection of a PAGA/pmIL-12 combination and naked pmIL-12, the levels of mIL-12 p70 and induced cytokines were assessed using ELISA in cultured tumor supernatants from CT-26 subcutaneous tumor-bearing BALB/c female mice. Compared to the bare pmIL-12, PAGA alone, and 5% glucose injection groups, the levels of IL-12, IFN-gamma, TNF-, and NO of PAGA/pmIL-12 group were increased. Immunohistochemistry revealed that the PAGA/pmIL-12 combination contained more natural killer (NK) cells, CD4(+) T cells, and antigen-presenting cells, such as macrophages and dendritic cells, than did bare pmIL-12 [81]. Moreover, the severity of insulitis in NOD mice can be decreased by the systemic delivery of pCAGGS mouse IL-10 expression plasmid/PAGA complexes. When compared to injections of naked DNA (34.5%) and untreated controls (90.9%), intravenous injections of the PAGA/DNA complex (PAGA) significantly decreased the frequency of severe insulitis in 12-week-old NOD mice (15.7%) [82].

- PVL poly(δ-valerolactone)

PVL-based polymers have been used as vectors for delivering genes. The transfection effectiveness was significantly impacted by the monomer arrangement and aliphatic side-chain length. He and colleagues produced new functional PVL copolymers using the ring opening polymerization of tertiary amine- and alkylated valerolactone-containing monomers. They proved that pDNA and functional PVL copolymers can successfully interact to generate pDNA/PVL polyplexes [83]. The transfection effectiveness of the best functional PVL copolymer with the ideal chemical structure can be 2.2 times greater than PEI [83].

- Aminated PAHA (aminated poly(α-hydroxy acids))

Due to their biocompatibility and biodegradability, amino-functionalized PAHAs offer a lot of potential for non-viral gene delivery. However, there have not been many reports of aminated PAHAs being used as gene delivery vehicles. This is because the cationic PAHAs that were initially employed were primarily made via condensation [84]. The resulting PAHAs typically had poorly regulated molecular weights, and were often somewhat wide in their molecular weight range. This was changed in the research of Cheng and colleagues [85]. They used an O-carboxyanhydride made from tyrosine to achieve ring-opening polymerization of 5-(4-(prop-2-yn-1-yloxy)benzyl)-1,3-dioxolane-2,4-dione (1), followed by sulfhydration of the alkyne function of 1 using 2-aminoethanethiol hydrochloride (AET). The final product, poly(1)-g-AET2 (PAET), a newly developed amino PAHA polymer, has low toxicity and outstanding cell penetration and gene delivery characteristics.

- PPE (polyphosphoester)

PPEs can be hydrolyzed through acid/base or enzymatically catalyzed processes [86]. The phosphoester linkages in polyphosphoesters can be broken down by enzymes under physiological circumstances. Alkaline phosphatases and phosphodiesterases are the two types of enzymes that can hydrolyze phosphodiester groups. The hydrolysis reaction, on the other hand, can be catalyzed in both acidic and basic environments, and the nature of the R groups, particularly the presence of functional acidic or basic side groups, may have an impact on the pace of deterioration. The degradation rate of PPE can be very slow compared to that of PBAE. As a result, PPE has potential uses in situations where long-term release is required. The PPE multilayers have significant uses in extended gene delivery and mouse osteoblast adhesion [87]. This enables the direct extension of gene expression in the presence of serum and continuous delivery of transcriptionally active DNA to mice osteoblasts, without the need of any external transfection agent. A layer-by-layer approach offers an alternate method for plasmid DNA binding that may be effective for the surface modification of implant materials or scaffolds for gene therapy and tissue regeneration. Such biodegradable multilayer assemblies are promising for the local and sustained delivery of plasmid DNA. Moreover, PPE-based micelle systems can bind siRNA and treat hypoxic tumors and lung cancer [88,89]. Chemotherapeutic agents, such as paclitaxel, can also be delivered through these micelles, and do not cause carrier-related toxicity nor activate innate immunity [88].

#### 2.1.3. Polylactide(Poly(lactic acid), PLA)

The main breakdown product of PLA degraded by hydrolysis and enzymatic activity is lactic acid, which is easily eliminated from the body, and has few negative effects [90]. As a result, PLA has become widely used in a variety of biomaterials for a variety of biological applications, such as sutures, scaffolds, surgical tools, and drug-delivery systems. Lacroix et al. developed reactive micelles constructed of polylactides as a dependable mRNA delivery system [91]. The transport and stability of mRNA were greatly enhanced by this approach. However, PLA itself has only seldom been used as a gene delivery vector, due to the scarcity of cationic compounds on its backbone. The allyl functional polylactide was developed to address this issue. Using allyl-functional lactide monomers, Cheng and associates produced allyl-functional polylactide by partially or fully converting allyl groups [92,93]. The newly created cationic polyactides (CPLAs) have been shown to be efficient at delivering siRNA and pDNA into cancer cells.

#### 2.1.4. Polycarbonates

Polycarbonates have been widely explored in numerous biomedical applications because of their highly tunable mechanical properties, as well as their biocompatibility, low toxicity, and biodegradability via hydrolysis in vivo. In contrast to the bulk degradation pattern found with aliphatic polyesters, polycarbonates are destroyed in vivo via surface erosion [94]. Moreover, unlike polyester degradation, the degradation of polycarbonate will not result in increased acidity, which could be harmful to loaded drugs or healthy tissues. Therefore, polycarbonates have become a promising candidate for use as gene delivery vectors. Zhuo’s group developed polyethylenimine-grafted polycarbonates that produced high gene transfection in 293T cells at high polymer concentrations while causing moderate cytotoxicity [95]. Zhang et al. developed a biodegradable cationic siRNA carrier for pancreatic cancer therapy, using polycarbonates generated from carbon dioxide (CPCHC) [96]. According to in vitro research, CPCHCs are capable of efficiently preventing siRNA from being destroyed by RNase, and encouraging siRNA to escape from the endosomes for an extended period of time. Both pancreatic cancer cell lines (PANC-1 and MiaPaCa-2) had drastically reduced K-ras gene expression following treatment with CPCHC/siRNA NPs. The treatment causes the cells to enter an apoptotic pathway, and significantly inhibits cell growth and migration. These findings highlight the intriguing potential of siRNA treatments mediated by CPCHC for the treatment of pancreatic cancer.

#### 2.1.5. Polyurethanes (PUs)

PUs, or polyurethanes, are synthetic polymers with urethane (or carbamate) linkages (-NH-COO-) in their main chains [97]. PUs can have their material qualities customized to serve a wide range of functions due to the wide number of building blocks that can be put into them. It is significant that PUs can be produced in vast quantities and processed in a variety of ways. As a result of these benefits, PUs are frequently employed in the industry. Cationic polyurethanes can be used as non-viral vectors, since they have positive charges and possible biofeatures. One such non-viral vector for gene delivery is N,N-diethylethylenediamine-polyurethane (DEDA-PU), which contains tertiary amines in the backbone and side chains [98]. DEDA-PU has a transfection effectiveness that is comparable to PDMAEMA and much less cytotoxicity than PDMAEMA, allowing it to deliver plasmid DNA into 293T cells. This had to do with the biodegradable characteristics of PU. Degradation tests revealed that DEDA-PU broke down hydrolytically, with a half-life of roughly 60 h in a 20 mM HEPES buffer at pH 7.4. According to this research, DEDA-PU is a fascinating option for additional study, and has the potential to be a biodegradable poly-cation for gene delivery. Polyurethane short-branch polyethylenimine (PU-PEI), another potentially safer gene delivery system with high transfection efficiency in comparison to conventional virus systems, was applied to deliver plasmid-bearing microRNA [99]. The results showed that this microRNA delivery mode promoted the differentiation capacity of stem cells. 

#### 2.1.6. Conjugated Polymers (CPs)

Conjugated polymers (CPs) are a type of polymer with a delocalized electronic structure and π-conjugated backbones [100,101,102,103]. These polymers have excellent physicochemical properties in photoluminescence, and have been widely used in bioimaging. CPs can also be modified with different side chains for use in drug and gene delivery systems for cancer therapy. Some of the most commonly used CPs for gene delivery include poly(p-phenyleneethynylene), polythiophene, poly(fluorine-co-phenylene), and poly(p-phenylenevinylene). These polymers are biocompatible, and can bond to nucleic acids through electrostatic interactions, hydrophobic interactions, or hydrogen bonds. CPs have promising applications as multifunctional gene delivery vehicles, and can be tracked by fluorescence imaging. For example, PEI-grafted hyperbranched conjugated polyelectrolytes (HCPEs) have been used for gene delivery and real-time imaging [104]. HCPE-PEI conjugates have a strong ability to condense plasmid DNA with low cytotoxicity, and confocal fluorescence imaging enables precise observation of the uptake of HCPEs-DNA multimers.

### 2.2. Natural Polymers

Naturally occurring polymers synthesized from living organisms are classified as natural polymers. These polymers possess good biocompatibility and are readily available in large yields, making them advantageous for the development of natural polymers for biological applications. The following is an overview of the classification of natural cationic polymers.

#### 2.2.1. Protein

- Histone

**Figure 4:**
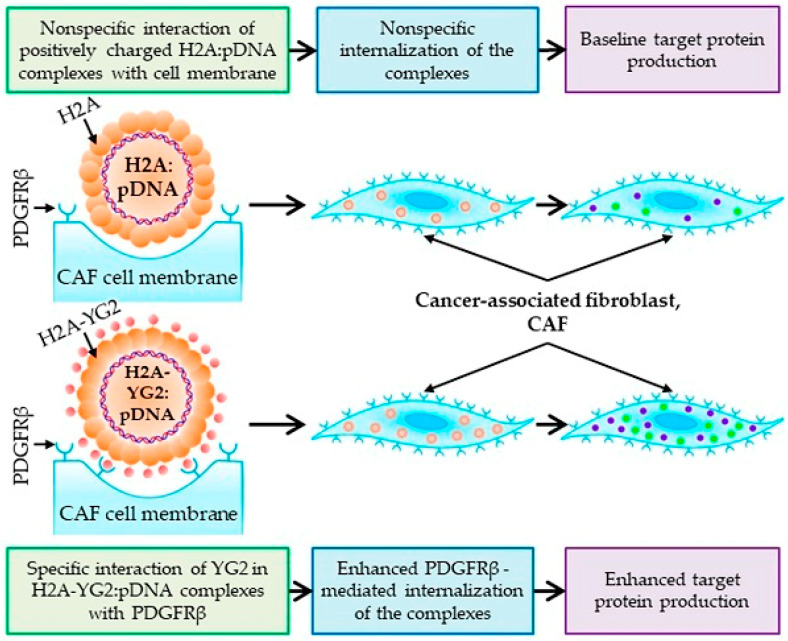
DNA carrier-containing histone for fibroblasts targeting [114].

Histones are the major protein components of chromatin. They serve as spools for DNA winding, and play important roles in gene regulation. Histones include H2A, H2B, H3 and H4, and H1, all of which have positively charged residues (lysine and arginine) and nuclear location signals (NLS) [105,106,107]. As a result, they are able to bind negatively charged nucleic acids through electrostatic interactions, and naturally target the nucleus [106]. The histone-mediated transfection of nucleic acids, called histonefection, is effective in transfecting DNA, mRNA, and siRNA, especially into primary cell lines [108]. Although the mechanism has not yet been investigated, histone entry into cells may not be achieved by endocytosis, as histones have been observed to cross cell membranes by passive diffusion [109]. Another advantage of histones as transfection reagents is their extremely low cytotoxicity. For instance, histone H1 and histone H1-like proteins have not been observed to be cytotoxic [110,111]. However, foreign histones are believed to affect the formation of nucleosomes by disrupting chronic histones and affecting normal replication and transcription [112,113]. Consequently, when histones are used in gene transfer studies, the interference of exogenous histones with the transcription of the underlying genes should be taken into account. A novel non-viral fibroblast-targeting DNA carrier called H2A-YG2 was made by fusing histone H2A with the PDGFR-binding peptide, YG2 (Figure 4) [114]. The fusion vector increased DNA internalization only in PDGFRβ-positive cells, indicating targeted gene delivery of the H2A-YG2 vector to PDGFRβ-positive tumor stromal cells.

Another gene delivery nanoplatform for cancer treatment is histone H2A-hybrided, upconversion luminescence (UCL)-guided mesoporous silica nanoparticles [UCNPs(BTZ)@mSiO_2_-H2A]. The H2A on the surface of the nanoparticles improves the biocompatibility, and enhances gene encapsulation as well as transfection efficiency. Using this platform, p53-null cancer cells underwent specific and efficient apoptosis related to p53 expression [115].

- Gelatin

Gelatin is extracted from animal collagen [116]. It is a biocompatible, biodegradable, and low-cost polymer. Gelatin is an amphiphilic polymer containing cationic and anionic charges as well as hydrophobic groups in a ratio of about 1:1:1 [116]. On the surface of gelatin are many reactive groups that are used to link and modify different molecules, giving the polymer a variety of functions. Unlike other polymers, gelatin has amino acid sequences in its structure, such as Arg-Gly-Asp (RGD) [117]. These amino acid sequences provide gelatin clear advantages over other polymers in terms of cell adhesion. Given its low charge density, the pH of the solution has a large impact on how charged the gelatin is [118]. Gelatin is, therefore, always cationized to enhance the polymer’s ability to bind with negatively charged DNA or cellular membranes for effective gene delivery. Polyethyleneimine, cholamine, ethylenediamine (EDA), putrescine, and spermidine are commonly used to introduce amine residues to the carboxyl groups of gelatin, in order to create cationized gelatin. Studies showed that siVEGF released from cationic gelatin microspheres inhibited the growth of tumors in vivo [119]. The suppression of siVEGF-transfected tumors was also accompanied by a significant reduction in the number of blood vessels. The complex of siVEGF with cationic gelatin microspheres was still detectable around the tumor ten days after injection, whereas free siVEGF had disappeared by this time [119]. Furthermore, compared to plasmid DNA condensed with non-cationic gelatin, adult osteoarticular chondrocytes expressed a 5-fold higher level of IGF when plasmid DNA was condensed with ethylenediamine-cationic gelatin [120]. Accordingly, cationized gelatin is a promising nucleic acid transfection reagent, especially for small RNA and plasmid DNA.

- Protamine

Protamine is a small polycationic peptide consisting of 50–110 amino acids, with a molecular weight of 4000–5000 Da. Due to electrostatic interactions between the positively charged protamine and the negatively charged nucleic acid-phosphate backbone, it binds to DNA during spermatogenesis and prevents it from degrading [121]. There are three types of protamine. The basic amino acid in monoprotamines is arginine, while diprotamines also include lysine or histidine, and triprotamines have all three. Sixty years ago, protamine was discovered as a carrier for RNA delivery to eukaryotic cells [122]. Today, by adjusting the concentration of salt, the ratio of protamine to RNA, and the concentration of the complex, it is possible to generate particles with average diameters specifically ranging from 50 nanometers (nm) to 1000 nm for different delivery needs [123]. 

The conventional siRNA/protamine particles exceeded 500 nm, and by preparing polyplex siRNA, a 120 nm multi-siRNA/protamine PECs particle was formed with protamine [124]. The complexes showed great stability, intracellular uptake, and biocompatibility. With the addition of chloroquine, the polysiRNA/protamine PECs successfully inhibited target gene expression in the breast cancer cell line MDA-MB-435, even in the presence of serum proteins. Chloroquine may have facilitated the process of siRNA release from endosomes.

Protamine not only facilitates delivery but also serves as an adjuvant to encourage antigen-presenting cell presentation and trigger antitumor immune responses. Mai et al. investigated the intranasal delivery of mRNA concentrated with positively charged protamine to form stable polycation–mRNA complexes, and encapsulated the complexes with DOTAP/Chol/DSPE-PEG cationic liposomes [125]. The cationic liposome/protamine complex (LPC) showed significant vaccine particle uptake efficiency, and enhanced the ability to stimulate dendritic cell maturation in vitro, which further induced a potent antitumor immune response. The intranasal immunization of mice by cationic LPC containing mRNA-encoding cytokeratin 19 elicited a strong cellular immune response and slowed tumor growth in an aggressive Lewis lung cancer model. The findings of this study show that cationic LPC-containing protamine can be used as a safe and effective adjuvant, and this mRNA preparation serves as a foundation for anti-cancer vaccination in humans.

#### 2.2.2. Polysaccharides

- Cyclodextrins

Cyclodextrins are naturally occurring cyclic oligosaccharides [126]. They consist of (α-1,4)-linked glucose units with a basket-like topology and an “endo-exo” amphipathic character. The complexes formed by unmodified cyclodextrins with plasmid DNA are unstable, and do not significantly enhance transfection efficiency [127]. Derivatives of cyclodextrins obtained by different modifications can significantly enhance the stability of the complexes and improve transfection efficiency. These modifications are usually attached to the 6-position of the glucose unit, and the modifying groups include pyridine amino, alkylimidazole, methoxyethylamino, or primary amine groups [127]. Another notable role of cyclodextrins in gene transfection is to reduce the cytotoxicity of other vectors. For example, the introduction of cyclodextrins in the polyamidines backbone can significantly reduce the cytotoxicity of the vector itself [128]. When cyclodextrins thread through specific polymer regions (main-chain complexes) or lateral chains (side-chain complexes), supramolecular assembly structures are created, such as cyclodextrins-based hydrogels [129]. When it comes to developing injectable hydrogels for cancer therapy, cyclodextrins-based hydrogels have been suggested as possible candidates because they offer sustained, long-lasting treatment at tumor locations while limiting exposure to non-target tissues. Complexation between α-cyclodextrins and cationic MPEG-PCL-PEI copolymer led to the formation of an injectable supramolecular hydrogel system. The resultant hydrogels were able to condense reporter plasmid DNA and release pDNA continuously for up to 7 days [130].

- Chitosan

**Figure 5:**
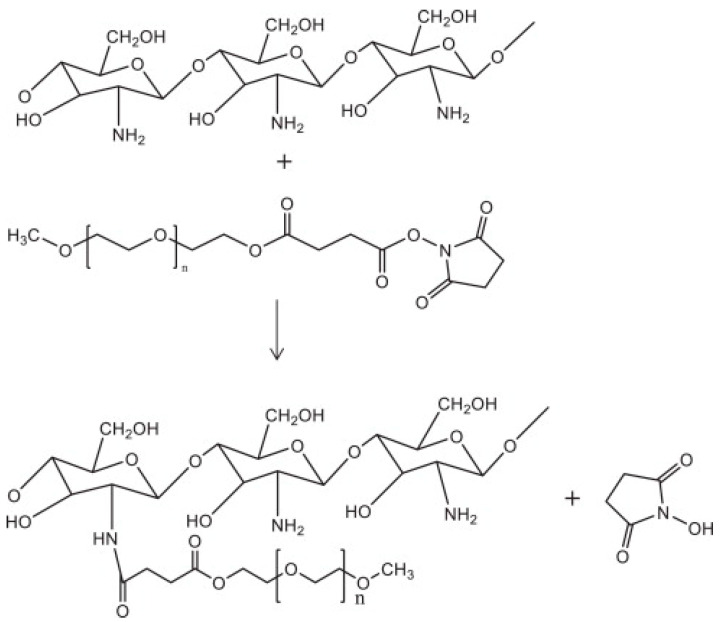
Synthesis of PEG-modified chitosan [132].

Chitin is a widely distributed substance in nature, found in the cell walls of fungi and in the exoskeletons of arthropods [131]. Its deacetylation product, chitosan, is a cationic polysaccharide with biocompatibility and biodegradability [131]. Due to its cationic nature, chitosan can form nanoparticles with negatively charged nucleic acids and mediate the intracellular transfection of nucleic acids. In addition, the amino group on the surface of chitosan allows for a proton sponge effect in the lysosome, which induces lysosomal escape. In an acidic environment, chitosan is soluble. In contrast, the solubility of chitosan becomes poor in a neutral or alkaline environment, a property that affects the application of chitosan as a gene transfection vector. There are some strategies to enhance its solubility. For example, the preparation of chitosan with a smaller molecular weight can significantly enhance its solubility, and chemical modifications such as PEGylated chitosan can also improve its solubility (Figure 5) [132]. An advantage of chitosan is its mucosal adhesion, which provides different routes of administration [133]. Intranasal administration has become more popular in human clinical studies, in part because of its lower invasiveness and toxicity. Chitosan nanocapsules were successfully administered in vivo intranasally, and showed successful siRNA transport from the nose to the brain for RNA interference (RNAi)-mediated galectin-1 knockdown in the GL261 mouse glioma line [134,135,136]. According to the data, chitosan-based nanocapsules may successfully transport nucleic acids to brain cancer cells, both in vitro and in vivo, by crossing the blood–brain barrier (BBB). 

Researchers have developed a nucleus-targeted co-delivery vector that can efficiently deliver genes and drugs directly into the nucleus of cancer cells. The vector is composed of grafted poly-(N-3-carbobenzyloxy-lysine) (CPCL), with a transactivator of transcription (TAT)-modified chitosan on the surface, and has been designed to achieve highly efficient nucleus-targeted gene and drug co-delivery. Confocal laser scanning microscopy (CLSM) has shown that the TAT-CPCL vector enters the nucleus more effectively than CPCL alone. By serving as a gene and drug co-delivery mechanism, the TAT-modified vector has demonstrated high gene transfection efficiency, high apoptosis, and low viability in HeLa cells [137]. 

## 3. The Applications of Cationic Polymers 

Gene therapy is the primary application of cationic polymers as transfection agents. By carrying plasmid DNA, mRNA, and siRNA, cationic polymers achieve therapeutically relevant functions such as gene augmentation, gene suppression, and genome editing. 

### 3.1. Gene Augmentation

- Protein replacement therapies

The most straightforward and perhaps simplest strategy for gene therapy involves the addition of a new protein-coding gene. For monogenic recessive diseases in which the disease-causing mutated gene is not functional, the therapeutic gene to be delivered will be the normal wild type of the gene. Delivery of the correct copy of the gene is expected to restore the production of the defective or missing protein, and thus restore the disease phenotype. 

A non-viral vector for oral insulin gene delivery was created by Lin et al. [138]. A copolymer of highly substituted branched chain polyethylenes (CS-g-bPEI) on chitosan makes up the vector, which helps the plasmid go through the intestinal epithelium, and prevents it from being destroyed by stomach acid. Such CS-g-bPEI/insulin plasmid DNA nanoparticles (NPs) enable systemic transgene expression for a number of days. In diabetic mice, a single dosage of oral NPs (600 micrograms of plasmatic insulin (pINS)) provided the animals with more than 10 days of protection from hyperglycemia. Similar hypoglycemia results were generated using a repeated dosage three times at 10-day intervals, with no liver injury noted.

- Vaccine

The large-scale use of mRNA vaccines in the context of the current epidemic has ignited a passionate interest in nucleic acid drugs. Theoretically, mRNA is capable of expressing any kind of protein; thus, it can be useful in the treatment of other diseases, in addition to preventing infectious diseases as a vaccine. Examples include the treatment of tumors, rare diseases, metabolic diseases, etc. The current mRNA delivery technology is based on a lipid nanoparticle platform, and the patents for this technology are in the hands of a few companies. Moreover, mRNA vaccines composed of lipid nanoparticles should be stored and transported at ultralow temperatures, severely limiting the use of vaccines in areas with high temperatures or limited conditions. Therefore, cationic polymers are uniquely suited as an alternative to liposomal nanoparticles.

**Figure 6:**
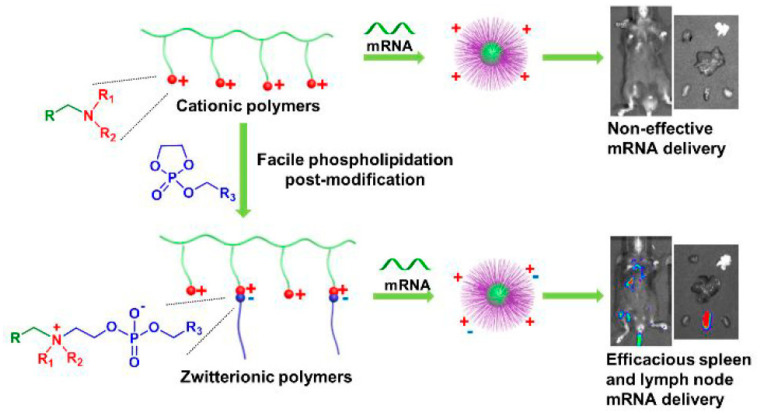
Systemic mRNA delivery to spleen and lymph nodes facilitated by the zwitterionic phospholipidation of cationic polymers. Reprinted with permission from Ref. [139]. Copyright 2021, American Chemical Society.

Liu et al. delivered mRNA to the spleen and lymph nodes in vivo via alkylated dioxophosphoryl oxides to cationic phospholipidated polymers (ZPPs) (Figure 6) [139]. This modular post-modification approach readily produced tunable amphiphilic species for antiserum purposes and simultaneously introduced alkyl chains to enhance endosomal escape, thereby transforming defective cationic polymers into effective amphiphilic mRNA carriers without the need for elaborate syntheses of functional monomers. ZPPs mediated 39,500-fold higher protein expression in vitro than their cationic parent, and selectively delivered effective mRNA into the spleen and lymph nodes after intravenous administration in vivo. 

Nanoparticles often show significant adjuvant effects in vaccine delivery. Cationic polymers, including PEI, polylysine, cationic dextran, and cationic gelatin, have been reported to exhibit a strong stimulation of Th1 responses characterized by the induction of CD4(+) T-cell proliferation and the secretion of Th1-related cytokines [140]. In addition, cationic polymers strongly inhibit LPS-induced TNF-α secretion from macrophages. The stimulatory ability of a cationic polymer is related to its degree of cationicity, and neutralization of a cationic polymer with an anionic polymer can completely abolish the stimulatory effect. The molecular weight of the polymer also affects its stimulatory ability, with larger molecules implying greater stimulatory ability. 

In addition to mRNA vaccines, DNA vaccines are also good options. With the aid of polyglucose, spermine (PG) conjugates, and fourth-generation polyamide dendrimers (PAMAM G4), researchers concentrated on ways to administer synthetic T-cell immunogens as DNA vaccines [141]. They improved the PG and PAMAM G4 complexes’ size, motility, and surface charge before testing the vaccine designs’ immunogenicity in BALB/c mice. As a result of being packaged in both the PG and the PAMAM G4 envelopes, the DNA vaccine’s immunogenicity increased, according to the findings. The strongest T-cell responses were seen in mice that were administered DNA vaccination complexes coated with PG, and these responses were noticeably higher than those shown in the group of animals that were prescribed the naked DNA combination and the DNA combination coated with PAMAM 4G.

### 3.2. Gene Suppression

- Small interfering RNA (siRNA)

Small interfering RNA (siRNA) is an initiator of RNA interference that stimulates the silencing of target mRNAs complementary to it, which is important for gene regulation and disease treatment. After entering the cytoplasm, siRNA is either loaded directly onto RISC or enters the Dicer-mediated interference process [142,143]. The activated RISC localizes to the homologous mRNA transcript by base-pairing, and cleaves the mRNA at a position 12 nt from the 3’ end of the siRNA to achieve gene silencing. Chemically synthesized siRNAs have the advantages of easy operation, high transfection efficiency, and low toxic effects on cells or tissues; moreover, they can be prepared on a large scale. These advantages are especially suitable for screening effective fragments of siRNAs under the uncertainty of gene target sites.

**Figure 7:**
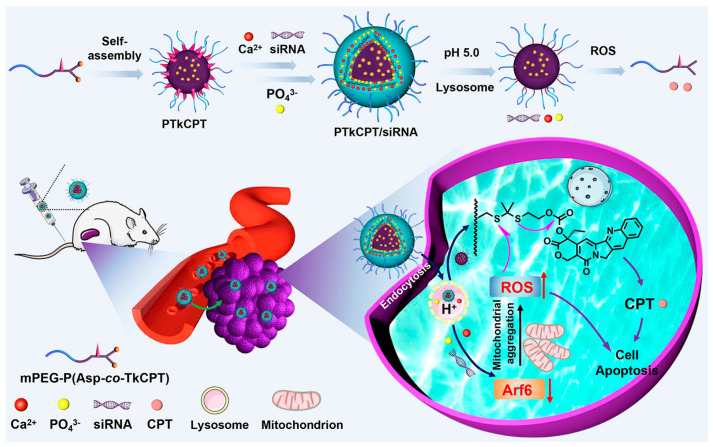
Oxidative stress and cascaded chemotherapy induced by RNAi through a polymer-calcium phosphate nanocapsule delivery system. Reprinted with permission from Ref. [144]. Copyright 2021, Elsevier.

Huang et al. combined siRNA and chemotherapeutic agents in the same nanoparticle to achieve in situ-activated ROS and CPT-based cascade therapy (Figure 7) [144]. They synthesized the copolymer mPEG-P (Asp-co-TkCPT) (PTkCPT) by covalently linking the hydrophobic chemotherapeutic drug CPT to the side chain of poly(ethylene glycol)-block poly(aspartic acid) (PEG-PAsp) via the dithione bond (Tk) of ROS-labile. PTkCPT was then self-assembled into a micelle containing a large number of -COOH groups in the micelle interlayer, and was used as a nanotemplate for CaP mineralization. In addition, Arf6 siRNA was loaded in the CaP layer. When PTkCPT/siRNA nanocapsules were endocytosed into cancer cells, the acidity of lysosomes may cause degradation of the CaP layer, and thus promote the release of siRNA. Arf6 siRNA blocked the Arf6 signaling pathway and promoted mitochondrial aggregation and subsequent ROS surge. ROS not only directly killed cancer cells, but also triggered chemotherapy by breaking the dithione bond in combination with CPT. Through this in situ cascade process, the nanoparticles significantly inhibited tumor growth in mice, with minimal side effects.

- Short hairpin RNA (shRNA)

shRNA technologies are DNA-based. Most shRNAs are transcribed through plasmid vectors, and shRNA-based therapies are, therefore, dependent on the delivery of plasmid DNA. The transcription of shRNA is typically driven by the U6 promoter, which drives high levels of constitutive expression, or by the weaker H1 promoter. After transcription, the shRNA sequence is recognized by an endogenous enzyme, Dicer, which processes the shRNA into siRNA duplexes. As with exogenously synthesized siRNA oligonucleotides, this endogenously produced siRNA binds to the target mRNA and is incorporated into the RISC complex for degradation of the target mRNA. A major advantage of shRNAs over siRNA systems is that shRNAs can be designed to be inducible. Another advantage of shRNAs is that their expressing sequences can be integrated into the genome and cause persistent silencing of the target gene.

**Figure 8:**
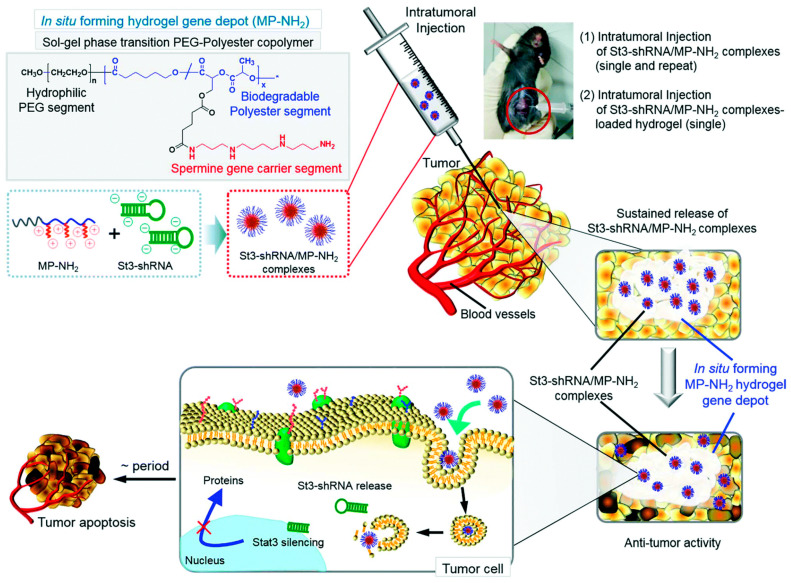
The injectable in situ-forming hydrogel gene depot used for improving the therapeutic effect of STAT3 shRNA. Reprinted with permission from Ref. [145]. Copyright 2021, The Royal Society of Chemistry.

Kim et al. designed biodegradable (methoxy)poly(ethylene glycol)-b-(polycaprolactone-poly(lactic acid)) copolymers (MP) that derive spermine groups with cationic properties (MP-NH_2_) at the pendant position of the MP chain (Figure 8) [145]. MP-NH_2_ can act as a gene carrier for St3-shRNA by forming electrostatic complexes with cationic spermine. This increases the stability of the complexes due to the protective effect of PEG in the biological environment, and can exhibit a sol-gel phase transition near body temperature, resulting in an intra-tumor injected MP-NH_2_ hydrogel library for the storage of St3-shRNAb. These complexes are not affected by external biomolecules, such as serum, DNase, and heparin, for a relatively long period of time (≥72 h). Intra-tumoral injections and St3-shRNA/MP-NH_2_ complex-loaded hydrogels showed prolonged and effective antitumor effects due to Stat3 knockdown.

### 3.3. Genome Editing

- CRISPR/Cas

**Figure 9:**
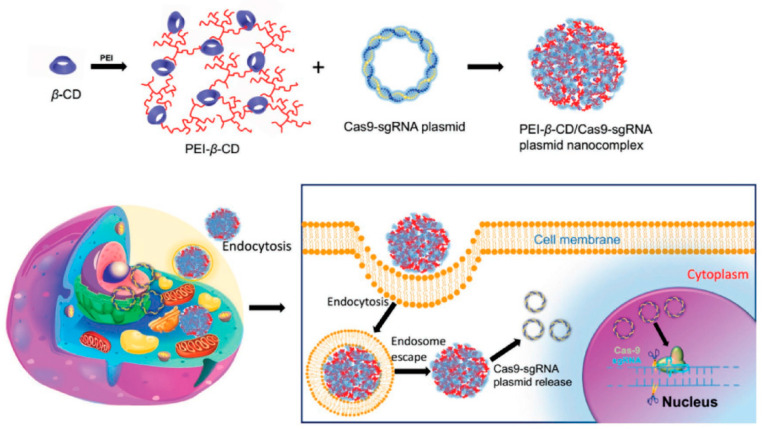
Cationic polymer used as CRISPR/Cas9 plasmid delivery vehicle for genome editing Reprinted with permission from Ref. [146]. Copyright 2018, WILEY-VCH Verlag GmbH & Co. KGaA.

The field of genome editing has undergone a revolution, thanks to the discovery of CRISPR/Cas. The CRIPSR/Cas component of the bacterial immune system causes genome-wide double-stranded DNA breaks, and facilitates gene editing by internal DNA repair processes. It has been noted that the cationic polymer polyethylenediamine-cyclodextrin (PC) facilitates the effective delivery of plasmids encoding Cas9 and sgRNA (Figure 9) [146]. When large plasmids are delivered by PC, they can coalesce and enclose them at high N/P ratios; this effectively edits two genomic loci: hemoglobin subunit beta (19.1%) and rhomboid 5 homolog 1 (RHBDF1 (7.0%)). Researchers developed macrophage-specific promoter-driven Cas9 expression plasmids (pM458 and pM330), and encapsulated them in cationic lipid-assisted PEG-b-PLGA nanoparticles to solve the problem of being unable to execute precise gene editing in target tissues and cells (CLAN) [147]. Due to the CD68 promoter’s unique production of Cas9, the Ntn1 gene was not disrupted in other cells. This method offers additional options for precise CRISPR/Cas9 in vivo gene editing. In vitro and in vivo, the CRISPR/Cas9 plasmid-encapsulated nanoparticles were able to exclusively express Cas9 in macrophages and their progenitor monocytes. More significantly, the resulting CLAN pM330/sgNtn1 successfully disrupted in vivo the expression of Ntn1 in macrophages and their Ntn1 gene in precursor monocytes, thereby reducing the expression of netrin-1 (encoded by Ntn1) and subsequently improving type 2 diabetes (T2D) symptoms.

Scientists have discovered that a related CRISPR system uses an enzyme called Cas13 that recognizes and cuts RNA instead of DNA [148]. Among its other functions, RNA acts as a messenger, passing instructions between DNA and cellular machinery to make proteins. The ability to edit RNA before it is translated into proteins could open up new therapeutic options for human diseases. Recently, researchers have used PBAE-based polymers to deliver Cas13a mRNA and guide RNA into the respiratory tracts of mice and hamsters for severe acute respiratory syndrome coronavirus 2 (SARS-CoV-2) treatment by nebulization [149]. In the study, mRNA-encoding Cas13a was used to attenuate influenza A virus and SARS-CoV-2 infections in mice and hamsters, respectively. The researchers designed CRISPR RNAs (crRNAs) that targeted highly conserved regions of influenza viruses PB1 and PB2, and CRISPR RNAs targeting the replicase and nucleocapsid genes of SARS-CoV-2. Polymer-formulated Cas13a mRNA and validated guide RNA were delivered into the respiratory tract via a nebulizer. Results showed that in mice, Cas13a effectively degraded influenza RNA in lung tissue upon post-infection delivery, while in hamsters, Cas13a delivery reduced SARS-CoV-2 replication and alleviated symptoms.

## 4. Concluding Remarks and Perspectives

In general, cationic polymer-based gene therapy shows great potential in clinical trials, but as research is still in its early stages, most studies are currently in pre-clinical phases. Table S2 outlines examples of cationic polymers used as gene delivery vehicles, with PEI being the most commonly used carrier according to data from ClinicalTrials.gov. These clinical trials aim to evaluate the safety, effectiveness, and feasibility of PEI in gene therapy, and determine optimal dosages and treatment plans. Meanwhile, protamine has been used clinically for many years, and is being studied for use as an RNA carrier in cancer immunotherapy. Trials have found it to be well-tolerated, with skin irritation and flu-like symptoms being the most common side effects. All protamine-mRNA vaccines induced appropriate immune responses, but further optimization may be necessary; combining the system with other anti-cancer therapies could improve their treatment efficacy. One of the main challenges in developing nucleic acid therapeutics is the body’s immune response, in addition to their short lifespans in the bloodstream. Another challenge is their premature release due to fast secretion. Fortunately, one of the advantages of cationic polymers is that they can be modified with different functional groups and ligands, which can slow down their clearance and provide specificity and selectivity for certain cell types or disease states. PEGylation can reduce the immunogenicity, stability, and retention time of nucleic acid delivery systems in the bloodstream [16]. Modification with lactate and folate can target salivary glycoprotein and folate receptors, which are often overexpressed in cancer cells [150,151,152,153]. Muscle peptide cationic polymers can be used for gene immunization at lower antigen levels; they do not require adjuvants, as they have demonstrated efficient gene delivery that can stimulate antigen-presenting cells and subsequent immune responses [154]. In addition, adding hydrophobic chains to cationic polymers is becoming increasingly popular to further improve gene delivery efficiency [155]. This is mainly because the resulting amphiphilic cationic derivatives are endowed with hydrophobicity, and hydrophobic chains are more easily absorbed by cells with lipophilic cell membranes.

Although cationic polymers provide a variety of benefits that make it possible to develop multifunctional carriers with less toxicity, their impact on cell membrane stability is still a factor that must be taken into consideration [156]. When cationic nanoparticles interact with cellular and nuclear membranes, they change the porosity and potential of the membranes, raise the intracellular calcium concentration, and cause inflammatory reactions. As a result, additional research is still needed to create appropriate cationic polymers for the delivery of nucleic acid drugs.

## Data Availability

Not applicable.

## Supplementary Materials

The following supporting information can be downloaded at: https://www.mdpi.com/article/10.3390/pharmaceutics15051502/s1, Table S1: Chemical structures of different cationic polymers; Table S2: Clinical trials with cationic polymers.

## References

[1] Chong Z.X., Yeap S.K., Ho W.Y. (2021). Transfection types, methods and strategies: A technical review. PeerJ.

[2] Dana H., Chalbatani G.M., Mahmoodzadeh H., Karimloo R., Rezaiean O., Moradzadeh A., Mehmandoost N., Moazzen F., Mazraeh A., Marmari V. (2017). Molecular Mechanisms and Biological Functions of siRNA. Int. J. Biomed. Sci..

[3] Oh Y.K., Park T.G. (2009). siRNA delivery systems for cancer treatment. Adv. Drug Deliv. Rev..

[4] Soutschek J., Akinc A., Bramlage B., Charisse K., Constien R., Donoghue M., Elbashir S., Geick A., Hadwiger P., Harborth J. (2004). Therapeutic silencing of an endogenous gene by systemic administration of modified siRNAs. Nature.

[5] Mottaghitalab F., Rastegari A., Farokhi M., Dinarvand R., Hosseinkhani H., Ou K.L., Pack D.W., Mao C., Dinarvand M., Fatahi Y. (2017). Prospects of siRNA applications in regenerative medicine. Int. J. Pharm..

[6] Pardi N., Hogan M.J., Porter F.W., Weissman D. (2018). mRNA vaccines—A new era in vaccinology. Nat. Rev. Drug Discov..

[7] Dolgin E. (2021). The tangled history of mRNA vaccines. Nature.

[8] Wang H., Feng Z., Xu B. (2019). Supramolecular Assemblies of Peptides or Nucleopeptides for Gene Delivery. Theranostics.

[9] Lundstrom K. (2018). Viral Vectors in Gene Therapy. Diseases.

[10] Bessis N., GarciaCozar F.J., Boissier M.C. (2004). Immune responses to gene therapy vectors: Influence on vector function and effector mechanisms. Gene Ther..

[11] Zu H., Gao D. (2021). Non-viral Vectors in Gene Therapy: Recent Development, Challenges, and Prospects. AAPS J..

[12] Pack D.W., Hoffman A.S., Pun S., Stayton P.S. (2005). Design and development of polymers for gene delivery. Nat. Rev. Drug Discov..

[13] Degors I.M.S., Wang C., Rehman Z.U., Zuhorn I.S. (2019). Carriers Break Barriers in Drug Delivery: Endocytosis and Endosomal Escape of Gene Delivery Vectors. Acc. Chem. Res..

[14] Vermeulen L.M.P., De Smedt S.C., Remaut K., Braeckmans K. (2018). The proton sponge hypothesis: Fable or fact?. Eur. J. Pharm. Biopharm..

[15] Shim M.S., Wang X., Ragan R., Kwon Y.J. (2010). Dynamics of nucleic acid/cationic polymer complexation and disassembly under biologically simulated conditions using in situ atomic force microscopy. Microsc. Res. Tech..

[16] Chen C., Yang Z., Tang X. (2018). Chemical modifications of nucleic acid drugs and their delivery systems for gene-based therapy. Med. Res. Rev..

[17] Li J., Liu L., Feng Z., Wang X., Huang Y., Dai H., Zhang L., Song F., Wang D., Zhang P. (2020). Tumor markers CA15-3, CA125, CEA and breast cancer survival by molecular subtype: A cohort study. Breast Cancer.

[18] McCutchan J.H., Pagano J.S. (1968). Enchancement of the infectivity of simian virus 40 deoxyribonucleic acid with diethylaminoethyl-dextran. J. Natl. Cancer Inst..

[19] Vaheri A., Pagano J.S. (1965). Infectious poliovirus RNA: A sensitive method of assay. Virology.

[20] Siewert C., Haas H., Nawroth T., Ziller A., Nogueira S.S., Schroer M.A., Blanchet C.E., Svergun D.I., Radulescu A., Bates F. (2019). Investigation of charge ratio variation in mRNA-DEAE-dextran polyplex delivery systems. Biomaterials.

[21] Uchiumi F., Watanabe T., Tanuma S. (2010). Characterization of various promoter regions of the human DNA helicase-encoding genes and identification of duplicated ets (GGAA) motifs as an essential transcription regulatory element. Exp. Cell Res..

[22] Felgner P.L., Gadek T.R., Holm M., Roman R., Chan H.W., Wenz M., Northrop J.P., Ringold G.M., Danielsen M. (1987). Lipofection: A highly efficient, lipid-mediated DNA-transfection procedure. Proc. Natl. Acad. Sci. USA.

[23] Thompson C.D., Frazier-Jessen M.R., Rawat R., Nordan R.P., Brown R.T. (1999). Evaluation of methods for transient transfection of a murine macrophage cell line, RAW 264.7. Biotechniques.

[24] Fischer D., Li Y., Ahlemeyer B., Krieglstein J., Kissel T. (2003). In vitro cytotoxicity testing of polycations: Influence of polymer structure on cell viability and hemolysis. Biomaterials.

[25] Vizcarra J.A., Karges S.L., Wettemann R.P. (2012). Immunization of beef heifers against gonadotropin-releasing hormone prevents luteal activity and pregnancy: Effect of conjugation to different proteins and effectiveness of adjuvants. J. Anim. Sci..

[26] Piedrafita D., Preston S., Kemp J., de Veer M., Sherrard J., Kraska T., Elhay M., Meeusen E. (2013). The effect of different adjuvants on immune parameters and protection following vaccination of sheep with a larval-specific antigen of the gastrointestinal nematode, Haemonchus contortus. PLoS ONE.

[27] Kwoh D.Y., Coffin C.C., Lollo C.P., Jovenal J., Banaszczyk M.G., Mullen P., Phillips A., Amini A., Fabrycki J., Bartholomew R.M. (1999). Stabilization of poly-L-lysine/DNA polyplexes for in vivo gene delivery to the liver. Biochim. Biophys. Acta.

[28] Wu G.Y., Wu C.H. (1987). Receptor-mediated in vitro gene transformation by a soluble DNA carrier system. J. Biol. Chem..

[29] Cotten M., Langle-Rouault F., Kirlappos H., Wagner E., Mechtler K., Zenke M., Beug H., Birnstiel M.L. (1990). Transferrin-polycation-mediated introduction of DNA into human leukemic cells: Stimulation by agents that affect the survival of transfected DNA or modulate transferrin receptor levels. Proc. Natl. Acad. Sci. USA.

[30] Mislick K.A., Baldeschwieler J.D., Kayyem J.F., Meade T.J. (1995). Transfection of folate-polylysine DNA complexes: Evidence for lysosomal delivery. Bioconjug. Chem..

[31] Farber F.E., Melnick J.L., Butel J.S. (1975). Optimal Conditions for Uptake of Exogenous DNA by Chinese-Hamster Lung-Cells Deficient in Hypoxanthine-Guanine Phosphoribosyltransferase. Biochim. Et Biophys. Acta.

[32] Ramsay E., Gumbleton M. (2002). Polylysine and polyornithine gene transfer complexes: A study of complex stability and cellular uptake as a basis for their differential in-vitro transfection efficiency. J. Drug Target..

[33] Li J., Liang H., Liu J., Wang Z. (2018). Poly (amidoamine) (PAMAM) dendrimer mediated delivery of drug and pDNA/siRNA for cancer therapy. Int. J. Pharm..

[34] Wu J., Huang W., He Z. (2013). Dendrimers as carriers for siRNA delivery and gene silencing: A review. Sci. World J..

[35] Boussif O., Lezoualc’h F., Zanta M.A., Mergny M.D., Scherman D., Demeneix B., Behr J.P. (1995). A versatile vector for gene and oligonucleotide transfer into cells in culture and in vivo: Polyethylenimine. Proc. Natl. Acad. Sci. USA.

[36] Haensler J., Szoka F.C. (1993). Polyamidoamine cascade polymers mediate efficient transfection of cells in culture. Bioconjug. Chem..

[37] Kukowska-Latallo J.F., Bielinska A.U., Johnson J., Spindler R., Tomalia D.A., Baker J.R. (1996). Efficient transfer of genetic material into mammalian cells using Starburst polyamidoamine dendrimers. Proc. Natl. Acad. Sci. USA.

[38] Monteagudo S., Perez-Martinez F.C., Perez-Carrion M.D., Guerra J., Merino S., Sanchez-Verdu M.P., Cena V. (2012). Inhibition of p42 MAPK using a nonviral vector-delivered siRNA potentiates the anti-tumor effect of metformin in prostate cancer cells. Nanomedicine.

[39] Yemul O., Imae T. (2005). Covalent-bonded immobilization of lipase on poly(phenylene sulfide) dendrimers and their hydrolysis ability. Biomacromolecules.

[40] Hollins A.J., Omidi Y., Benter I.F., Akhtar S. (2007). Toxicogenomics of drug delivery systems: Exploiting delivery system-induced changes in target gene expression to enhance siRNA activity. J. Drug Target..

[41] Bugno J., Hsu H.J., Pearson R.M., Noh H., Hong S. (2016). Size and Surface Charge of Engineered Poly(amidoamine) Dendrimers Modulate Tumor Accumulation and Penetration: A Model Study Using Multicellular Tumor Spheroids. Mol. Pharm..

[42] Kaur D., Jain K., Mehra N.K., Kesharwani P., Jain N.K. (2016). A review on comparative study of PPI and PAMAM dendrimers. J. Nanopart. Res..

[43] Hashemi M., Tabatabai S.M., Parhiz H., Milanizadeh S., Amel Farzad S., Abnous K., Ramezani M. (2016). Gene delivery efficiency and cytotoxicity of heterocyclic amine-modified PAMAM and PPI dendrimers. Mater. Sci. Eng. C Mater. Biol. Appl..

[44] Liu H., Wang Y., Wang M., Xiao J., Cheng Y. (2014). Fluorinated poly(propylenimine) dendrimers as gene vectors. Biomaterials.

[45] Yue Y., Jin F., Deng R., Cai J., Dai Z., Lin M.C., Kung H.F., Mattebjerg M.A., Andresen T.L., Wu C. (2011). Revisit complexation between DNA and polyethylenimine--effect of length of free polycationic chains on gene transfection. J. Control. Release.

[46] Choudhury C.K., Kumar A., Roy S. (2013). Characterization of conformation and interaction of gene delivery vector polyethylenimine with phospholipid bilayer at different protonation state. Biomacromolecules.

[47] Nguyen J., Szoka F.C. (2012). Nucleic acid delivery: The missing pieces of the puzzle?. Acc. Chem. Res..

[48] Ziebarth J.D., Wang Y. (2010). Understanding the protonation behavior of linear polyethylenimine in solutions through Monte Carlo simulations. Biomacromolecules.

[49] Itaka K., Harada A., Yamasaki Y., Nakamura K., Kawaguchi H., Kataoka K. (2004). In situ single cell observation by fluorescence resonance energy transfer reveals fast intra-cytoplasmic delivery and easy release of plasmid DNA complexed with linear polyethylenimine. J. Gene Med..

[50] Wightman L., Kircheis R., Rossler V., Carotta S., Ruzicka R., Kursa M., Wagner E. (2001). Different behavior of branched and linear polyethylenimine for gene delivery in vitro and in vivo. J. Gene Med..

[51] Zhang P., Wagner E. (2017). History of Polymeric Gene Delivery Systems. Top. Curr. Chem..

[52] Wen Y., Pan S., Luo X., Zhang X., Zhang W., Feng M. (2009). A biodegradable low molecular weight polyethylenimine derivative as low toxicity and efficient gene vector. Bioconjug. Chem..

[53] Teo P.Y., Yang C., Hedrick J.L., Engler A.C., Coady D.J., Ghaem-Maghami S., George A.J., Yang Y.Y. (2013). Hydrophobic modification of low molecular weight polyethylenimine for improved gene transfection. Biomaterials.

[54] Zintchenko A., Philipp A., Dehshahri A., Wagner E. (2008). Simple modifications of branched PEI lead to highly efficient siRNA carriers with low toxicity. Bioconjug. Chem..

[55] Jiang H.L., Islam M.A., Xing L., Firdous J., Cao W., He Y.J., Zhu Y., Cho K.H., Li H.S., Cho C.S. (2017). Degradable Polyethylenimine-Based Gene Carriers for Cancer Therapy. Top. Curr. Chem..

[56] Cherng J.Y., van de Wetering P., Talsma H., Crommelin D.J., Hennink W.E. (1996). Effect of size and serum proteins on transfection efficiency of poly ((2-dimethylamino)ethyl methacrylate)-plasmid nanoparticles. Pharm. Res..

[57] van de Wetering P., Cherng J.Y., Talsma H., Crommelin D.J.A., Hennink W.E. (1998). 2-(dimethylamino)ethyl methacrylate based (co)polymers as gene transfer agents. J. Control. Release.

[58] Jones R.A., Poniris M.H., Wilson M.R. (2004). pDMAEMA is internalised by endocytosis but does not physically disrupt endosomes. J. Control. Release.

[59] Zuidam N.J., Posthuma G., de Vries E.T., Crommelin D.J., Hennink W.E., Storm G. (2000). Effects of physicochemical characteristics of poly(2-(dimethylamino)ethyl methacrylate)-based polyplexes on cellular association and internalization. J. Drug Target.

[60] Funhoff A.M., van Nostrum C.F., Lok M.C., Kruijtzer J.A., Crommelin D.J., Hennink W.E. (2005). Cationic polymethacrylates with covalently linked membrane destabilizing peptides as gene delivery vectors. J. Control. Release.

[61] Lynn D.M., Anderson D.G., Putnam D., Langer R. (2001). Accelerated discovery of synthetic transfection vectors: Parallel synthesis and screening of a degradable polymer library. J. Am. Chem. Soc..

[62] Lynn D.M., Langer R. (2000). Degradable Poly(β-amino esters):  Synthesis, Characterization, and Self-Assembly with Plasmid DNA. J. Am. Chem. Soc..

[63] Gong J.H., Wang Y., Xing L., Cui P.F., Qiao J.B., He Y.J., Jiang H.L. (2018). Biocompatible fluorinated poly(beta-amino ester)s for safe and efficient gene therapy. Int. J. Pharm..

[64] Song W., Tang Z., Li M., Lv S., Yu H., Ma L., Zhuang X., Huang Y., Chen X. (2012). Tunable pH-sensitive poly(beta-amino ester)s synthesized from primary amines and diacrylates for intracellular drug delivery. Macromol. Biosci..

[65] Chen J., Ouyang J., Kong J., Zhong W., Xing M.M. (2013). Photo-cross-linked and pH-sensitive biodegradable micelles for doxorubicin delivery. ACS Appl. Mater. Interfaces.

[66] Liu M., Chen J., Xue Y.N., Liu W.M., Zhuo R.X., Huang S.W. (2009). Poly(beta-aminoester)s with pendant primary amines for efficient gene delivery. Bioconjug. Chem..

[67] Chen J., Huang S.W., Liu M., Zhuo R.X. (2007). Synthesis and degradation of poly(beta-aminoester) with pendant primary amine. Polymer.

[68] Cutlar L., Zhou D., Gao Y., Zhao T., Greiser U., Wang W., Wang W. (2015). Highly Branched Poly(beta-Amino Esters): Synthesis and Application in Gene Delivery. Biomacromolecules.

[69] Chen J., Wu C., Oupicky D. (2009). Bioreducible hyperbranched poly(amido amine)s for gene delivery. Biomacromolecules.

[70] Chen J., Zehtabi F., Ouyang J., Kong J.M., Zhong W., Xing M.M.Q. (2012). Reducible self-assembled micelles for enhanced intracellular delivery of doxorubicin. J. Mater. Chem..

[71] Tang S., Yin Q., Su J., Sun H., Meng Q., Chen Y., Chen L., Huang Y., Gu W., Xu M. (2015). Inhibition of metastasis and growth of breast cancer by pH-sensitive poly (beta-amino ester) nanoparticles co-delivering two siRNA and paclitaxel. Biomaterials.

[72] Tzeng S.Y., Green J.J. (2013). Subtle changes to polymer structure and degradation mechanism enable highly effective nanoparticles for siRNA and DNA delivery to human brain cancer. Adv. Healthc. Mater..

[73] Huang J.Y., Gao Y., Cutlar L., O’Keeffe-Ahern J., Zhao T., Lin F.H., Zhou D., McMahon S., Greiser U., Wang W. (2015). Tailoring highly branched poly(beta-amino ester)s: A synthetic platform for epidermal gene therapy. Chem. Commun..

[74] Cordeiro R.A., Serra A., Coelho J.F.J., Faneca H. (2019). Poly(beta-amino ester)-based gene delivery systems: From discovery to therapeutic applications. J. Control. Release.

[75] Fornaguera C., Guerra-Rebollo M., Angel Lazaro M., Castells-Sala C., Meca-Cortes O., Ramos-Perez V., Cascante A., Rubio N., Blanco J., Borros S. (2018). mRNA Delivery System for Targeting Antigen-Presenting Cells In Vivo. Adv. Healthc. Mater..

[76] Tzeng S.Y., Hung B.P., Grayson W.L., Green J.J. (2012). Cystamine-terminated poly(beta-amino ester)s for siRNA delivery to human mesenchymal stem cells and enhancement of osteogenic differentiation. Biomaterials.

[77] Liu S., Gao Y., Zhou D., Zeng M., Alshehri F., Newland B., Lyu J., O’Keeffe-Ahern J., Greiser U., Guo T. (2019). Highly branched poly(beta-amino ester) delivery of minicircle DNA for transfection of neurodegenerative disease related cells. Nat. Commun..

[78] Putnam D., Langer R. (1999). Poly(4-hydroxy-L-proline ester): Low-temperature polycondensation and plasmid DNA complexation. Macromolecules.

[79] Lim Y.B., Choi Y.H., Park J.S. (1999). A self-destroying polycationic polymer: Biodegradable poly(4-hydroxy-L-proline ester). J. Am. Chem. Soc..

[80] Lim Y.B., Han S.O., Kong H.U., Lee Y., Park J.S., Jeong B., Kim S.W. (2000). Biodegradable polyester, poly[alpha-(4-aminobutyl)-L-glycolic acid], as a non-toxic gene carrier. Pharm. Res..

[81] Maheshwari A., Han S., Mahato R.I., Kim S.W. (2002). Biodegradable polymer-based interleukin-12 gene delivery: Role of induced cytokines, tumor infiltrating cells and nitric oxide in anti-tumor activity. Gene Ther..

[82] Koh J.J., Ko K.S., Lee M., Han S., Park J.S., Kim S.W. (2000). Degradable polymeric carrier for the delivery of IL-10 plasmid DNA to prevent autoimmune insulitis of NOD mice. Gene Ther..

[83] Song L., Ding A.X., Zhang K.X., Gong B., Lu Z.L., He L. (2017). Degradable polyesters via ring-opening polymerization of functional valerolactones for efficient gene delivery. Org. Biomol. Chem..

[84] Basu A., Kunduru K.R., Katzhendler J., Domb A.J. (2016). Poly(alpha-hydroxy acid)s and poly(alpha-hydroxy acid-co-alpha-amino acid)s derived from amino acid. Adv. Drug Deliv. Rev..

[85] Zhang Z., Yin L., Xu Y., Tong R., Lu Y., Ren J., Cheng J. (2012). Facile functionalization of polyesters through thiol-yne chemistry for the design of degradable, cell-penetrating and gene delivery dual-functional agents. Biomacromolecules.

[86] Yilmaz Z.E., Jerome C. (2016). Polyphosphoesters: New Trends in Synthesis and Drug Delivery Applications. Macromol. Biosci..

[87] Lu Z.Z., Wu J., Sun T.M., Ji J., Yan L.F., Wang J. (2008). Biodegradable polycation and plasmid DNA multilayer film for prolonged gene delivery to mouse osteoblasts. Biomaterials.

[88] Sun T.M., Du J.Z., Yao Y.D., Mao C.Q., Dou S., Huang S.Y., Zhang P.Z., Leong K.W., Song E.W., Wang J. (2011). Simultaneous delivery of siRNA and paclitaxel via a “two-in-one” micelleplex promotes synergistic tumor suppression. ACS Nano.

[89] Mao C.Q., Xiong M.H., Liu Y., Shen S., Du X.J., Yang X.Z., Dou S., Zhang P.Z., Wang J. (2014). Synthetic lethal therapy for KRAS mutant non-small-cell lung carcinoma with nanoparticle-mediated CDK4 siRNA delivery. Mol. Ther..

[90] Tyler B., Gullotti D., Mangraviti A., Utsuki T., Brem H. (2016). Polylactic acid (PLA) controlled delivery carriers for biomedical applications. Adv. Drug Deliv. Rev..

[91] Lacroix C., Humanes A., Coiffier C., Gigmes D., Verrier B., Trimaille T. (2020). Polylactide-Based Reactive Micelles as a Robust Platform for mRNA Delivery. Pharm. Res..

[92] Chen C.K., Law W.C., Aalinkeel R., Nair B., Kopwitthaya A., Mahajan S.D., Reynolds J.L., Zou J., Schwartz S.A., Prasad P.N. (2012). Well-defined degradable cationic polylactide as nanocarrier for the delivery of siRNA to silence angiogenesis in prostate cancer. Adv. Healthc. Mater..

[93] Jones C.H., Chen C.K., Jiang M., Fang L., Cheng C., Pfeifer B.A. (2013). Synthesis of cationic polylactides with tunable charge densities as nanocarriers for effective gene delivery. Mol. Pharm..

[94] Ong Z.Y., Fukushima K., Coady D.J., Yang Y.Y., Ee P.L., Hedrick J.L. (2011). Rational design of biodegradable cationic polycarbonates for gene delivery. J. Control. Release.

[95] Wang C.F., Lin Y.X., Jiang T., He F., Zhuo R.X. (2009). Polyethylenimine-grafted polycarbonates as biodegradable polycations for gene delivery. Biomaterials.

[96] Segel M., Lash B., Song J., Ladha A., Liu C.C., Jin X., Mekhedov S.L., Macrae R.K., Koonin E.V., Zhang F. (2021). Mammalian retrovirus-like protein PEG10 packages its own mRNA and can be pseudotyped for mRNA delivery. Science.

[97] Cherng J.Y., Hou T.Y., Shih M.F., Talsma H., Hennink W.E. (2013). Polyurethane-based drug delivery systems. Int. J. Pharm..

[98] Yang T.F., Chin W.K., Cherng J.Y., Shau M.D. (2004). Synthesis of novel biodegradable cationic polymer: N,N-diethylethylenediamine polyurethane as a gene carrier. Biomacromolecules.

[99] Chien C.S., Wang C.Y., Yang Y.P., Chou S.J., Ko Y.L., Tsai F.T., Yu W.C., Chang C.C., Cherng J.Y., Yang M.Y. (2020). Using cationic polyurethane-short branch PEI as microRNA-driven nano-delivery system for stem cell differentiation. J. Chin. Med. Assoc..

[100] Yang G., Lv F., Wang B., Liu L., Yang Q., Wang S. (2012). Multifunctional non-viral delivery systems based on conjugated polymers. Macromol. Biosci..

[101] Song G., Lv F., Huang Y., Bai H., Wang S. (2022). Conjugated Polymers for Gene Delivery and Photothermal Gene Expression. Chempluschem.

[102] Wang X., He F., Li L., Wang H., Yan R., Li L. (2013). Conjugated oligomer-based fluorescent nanoparticles as functional nanocarriers for nucleic acids delivery. ACS Appl. Mater. Interfaces.

[103] Ahmed M.S., Dutta R.K., Manandhar P., Li X., Torabi H., Barrios A., Wang P., Chinnapaiyan S., Unwalla H.J., Moon J.H. (2019). A guanylurea-functionalized conjugated polymer enables RNA interference in ex vivo human airway epithelium. Chem. Commun..

[104] Wang G., Yin H., Ng J.C.Y., Cai L.P., Li J., Tang B.Z., Liu B. (2013). Polyethyleneimine-grafted hyperbranched conjugated polyelectrolytes: Synthesis and imaging of gene delivery. Polym. Chem..

[105] Kornberg R.D., Lorch Y. (1999). Twenty-five years of the nucleosome, fundamental particle of the eukaryote chromosome. Cell.

[106] Palau J., Climent F., Aviles F.J., Morros A., Soliva M. (1977). Interactions of histones and histone peptides with DNA Thermal denaturation and solubility studies. Biochim. Biophys. Acta.

[107] Baake M., Doenecke D., Albig W. (2001). Characterisation of nuclear localisation signals of the four human core histones. J. Cell. Biochem..

[108] Haberland A., Bottger M. (2005). Nuclear proteins as gene-transfer vectors. Biotechnol. Appl. Biochem..

[109] Hariton-Gazal E., Rosenbluh J., Graessmann A., Gilon C., Loyter A. (2003). Direct translocation of histone molecules across cell membranes. J. Cell. Sci..

[110] Budker V., Hagstrom J.E., Lapina O., Eifrig D., Fritz J., Wolff J.A. (1997). Protein/amphipathic polyamine complexes enable highly efficient transfection with minimal toxicity. Biotechniques.

[111] Zaitsev S.V., Haberland A., Otto A., Vorob’ev V.I., Haller H., Bottger M. (1997). H1 and HMG17 extracted from calf thymus nuclei are efficient DNA carriers in gene transfer. Gene Ther..

[112] Lorch Y., LaPointe J.W., Kornberg R.D. (1987). Nucleosomes inhibit the initiation of transcription but allow chain elongation with the displacement of histones. Cell.

[113] Kaouass M., Beaulieu R., Balicki D. (2006). Histonefection: Novel and potent non-viral gene delivery. J. Control. Release.

[114] Kuzmich A., Rakitina O., Didych D., Potapov V., Zinovyeva M., Alekseenko I., Sverdlov E. (2020). Novel Histone-Based DNA Carrier Targeting Cancer-Associated Fibroblasts. Polymers.

[115] Rong J., Li P., Ge Y., Chen H., Wu J., Zhang R., Lao J., Lou D., Zhang Y. (2020). Histone H2A-peptide-hybrided upconversion mesoporous silica nanoparticles for bortezomib/p53 delivery and apoptosis induction. Colloids Surf. B.

[116] Elzoghby A.O. (2013). Gelatin-based nanoparticles as drug and gene delivery systems: Reviewing three decades of research. J. Control. Release.

[117] Wang H., Boerman O.C., Sariibrahimoglu K., Li Y., Jansen J.A., Leeuwenburgh S.C. (2012). Comparison of micro- vs. nanostructured colloidal gelatin gels for sustained delivery of osteogenic proteins: Bone morphogenetic protein-2 and alkaline phosphatase. Biomaterials.

[118] Zwiorek K., Kloeckner J., Wagner E., Coester C. (2005). Gelatin nanoparticles as a new and simple gene delivery system. J. Pharm. Pharm. Sci..

[119] Matsumoto G., Kushibiki T., Kinoshita Y., Lee U., Omi Y., Kubota E., Tabata Y. (2006). Cationized gelatin delivery of a plasmid DNA expressing small interference RNA for VEGF inhibits murine squamous cell carcinoma. Cancer Sci..

[120] Xu X., Capito R.M., Spector M. (2008). Delivery of plasmid IGF-1 to chondrocytes via cationized gelatin nanoparticles. J. Biomed. Mater. Res. A.

[121] Jarzebska N.T., Mellett M., Frei J., Kundig T.M., Pascolo S. (2021). Protamine-Based Strategies for RNA Transfection. Pharmaceutics.

[122] Amos H. (1961). Protamine Enhancement of Rna Uptake by Cultured Chick Cells. Biochem. Biophys. Res. Commun..

[123] Tusup M., Pascolo S. (2017). Generation of Immunostimulating 130 nm Protamine-RNA nanoparticles. Methods Mol. Biol..

[124] Yoo H., Mok H. (2015). Evaluation of multimeric siRNA conjugates for efficient protamine-based delivery into breast cancer cells. Arch. Pharm. Res..

[125] Mai Y., Guo J., Zhao Y., Ma S., Hou Y., Yang J. (2020). Intranasal delivery of cationic liposome-protamine complex mRNA vaccine elicits effective anti-tumor immunity. Cell. Immunol..

[126] Crini G., Fourmentin S., Fenyvesi É., Torri G., Fourmentin M., Morin-Crini N. (2018). Cyclodextrins, from molecules to applications. Environ. Chem. Lett..

[127] Cryan S.A., Holohan A., Donohue R., Darcy R., O’Driscoll C.M. (2004). Cell transfection with polycationic cyclodextrin vectors. Eur. J. Pharm. Sci..

[128] Hwang S.J., Bellocq N.C., Davis M.E. (2001). Effects of structure of beta-cyclodextrin-containing polymers on gene delivery. Bioconjug. Chem..

[129] Liu J., Tian B., Liu Y., Wan J.B. (2021). Cyclodextrin-Containing Hydrogels: A Review of Preparation Method, Drug Delivery, and Degradation Behavior. Int. J. Mol. Sci..

[130] Liu X., Li Z., Loh X.J., Chen K., Li Z., Wu Y.L. (2019). Targeted and Sustained Corelease of Chemotherapeutics and Gene by Injectable Supramolecular Hydrogel for Drug-Resistant Cancer Therapy. Macromol. Rapid Commun..

[131] Elieh-Ali-Komi D., Hamblin M.R. (2016). Chitin and Chitosan: Production and Application of Versatile Biomedical Nanomaterials. Int. J. Adv. Res..

[132] Anraku M., Hiraga A., Iohara D., Uekama K., Tomida H., Otagiri M., Hirayama F. (2014). Preparation and antioxidant activity of PEGylated chitosans with different particle sizes. Int. J. Biol. Macromol..

[133] TM M.W., Lau W.M., Khutoryanskiy V.V. (2018). Chitosan and Its Derivatives for Application in Mucoadhesive Drug Delivery Systems. Polymers.

[134] Lara-Velazquez M., Alkharboosh R., Norton E.S., Ramirez-Loera C., Freeman W.D., Guerrero-Cazares H., Forte A.J., Quinones-Hinojosa A., Sarabia-Estrada R. (2020). Chitosan-Based Non-viral Gene and Drug Delivery Systems for Brain Cancer. Front. Neurol..

[135] Islam S.U., Shehzad A., Ahmed M.B., Lee Y.S. (2020). Intranasal Delivery of Nanoformulations: A Potential Way of Treatment for Neurological Disorders. Molecules.

[136] Posadas I., Monteagudo S., Cena V. (2016). Nanoparticles for brain-specific drug and genetic material delivery, imaging and diagnosis. Nanomedicine.

[137] Wang G.H., Cai Y.Y., Du J.K., Li L., Li Q., Yang H.K., Lin J.T. (2018). TAT-conjugated chitosan cationic micelle for nuclear-targeted drug and gene co-delivery. Colloids Surf. B Biointerfaces.

[138] Lin P.Y., Chiu Y.L., Huang J.H., Chuang E.Y., Mi F.L., Lin K.J., Juang J.H., Sung H.W., Leong K.W. (2018). Oral Nonviral Gene Delivery for Chronic Protein Replacement Therapy. Adv. Sci..

[139] Liu S., Wang X., Yu X., Cheng Q., Johnson L.T., Chatterjee S., Zhang D., Lee S.M., Sun Y., Lin T.C. (2021). Zwitterionic Phospholipidation of Cationic Polymers Facilitates Systemic mRNA Delivery to Spleen and Lymph Nodes. J. Am. Chem. Soc..

[140] Chen H., Li P., Yin Y., Cai X., Huang Z., Chen J., Dong L., Zhang J. (2010). The promotion of type 1 T helper cell responses to cationic polymers in vivo via toll-like receptor-4 mediated IL-12 secretion. Biomaterials.

[141] Karpenko L.I., Apartsin E.K., Dudko S.G., Starostina E.V., Kaplina O.N., Antonets D.V., Volosnikova E.A., Zaitsev B.N., Bakulina A.Y., Venyaminova A.G. (2020). Cationic Polymers for the Delivery of the Ebola DNA Vaccine Encoding Artificial T-Cell Immunogen. Vaccines.

[142] Sashital D.G., Doudna J.A. (2010). Structural insights into RNA interference. Curr. Opin. Struct. Biol..

[143] Piombo E., Vetukuri R.R., Broberg A., Kalyandurg P.B., Kushwaha S., Funck Jensen D., Karlsson M., Dubey M. (2021). Role of Dicer-Dependent RNA Interference in Regulating Mycoparasitic Interactions. Microbiol. Spectr..

[144] Huang J., Zheng C., Xiao H., Huang H., Wang Y., Lin M., Pang J., Wang Y., Yuan Y., Shuai X. (2021). A polymer--calcium phosphate nanocapsule for RNAi-induced oxidative stress and cascaded chemotherapy. J. Control. Release.

[145] Kim D.Y., Ju H.J., Kim J.H., Choi S., Kim M.S. (2021). Injectable in situ forming hydrogel gene depot to improve the therapeutic effect of STAT3 shRNA. Biomater. Sci..

[146] Zhang Z., Wan T., Chen Y., Chen Y., Sun H., Cao T., Songyang Z., Tang G., Wu C., Ping Y. (2019). Cationic Polymer-Mediated CRISPR/Cas9 Plasmid Delivery for Genome Editing. Macromol. Rapid Commun..

[147] Luo Y.L., Xu C.F., Li H.J., Cao Z.T., Liu J., Wang J.L., Du X.J., Yang X.Z., Gu Z., Wang J. (2018). Macrophage-Specific in Vivo Gene Editing Using Cationic Lipid-Assisted Polymeric Nanoparticles. ACS Nano.

[148] Cox D.B.T., Gootenberg J.S., Abudayyeh O.O., Franklin B., Kellner M.J., Joung J., Zhang F. (2017). RNA editing with CRISPR-Cas13. Science.

[149] Blanchard E.L., Vanover D., Bawage S.S., Tiwari P.M., Rotolo L., Beyersdorf J., Peck H.E., Bruno N.C., Hincapie R., Michel F. (2021). Treatment of influenza and SARS-CoV-2 infections via mRNA-encoded Cas13a in rodents. Nat. Biotechnol..

[150] Wang H., Wan G., Liu Y., Chen B., Chen H., Zhang S., Wang D., Xiong Q., Zhang N., Wang Y. (2016). Dual-responsive nanoparticles based on oxidized pullulan and a disulfide-containing poly(β-amino) ester for efficient delivery of genes and chemotherapeutic agents targeting hepatoma. Polym. Chem..

[151] Zhang S., Wang D., Li Y., Li L., Chen H., Xiong Q., Liu Y., Wang Y. (2018). pH- and redox-responsive nanoparticles composed of charge-reversible pullulan-based shells and disulfide-containing poly(ss-amino ester) cores for co-delivery of a gene and chemotherapeutic agent. Nanotechnology.

[152] Wang Y., Chen H., Liu Y., Wu J., Zhou P., Wang Y., Li R., Yang X., Zhang N. (2013). pH-sensitive pullulan-based nanoparticle carrier of methotrexate and combretastatin A4 for the combination therapy against hepatocellular carcinoma. Biomaterials.

[153] Kaneo Y., Tanaka T., Nakano T., Yamaguchi Y. (2001). Evidence for receptor-mediated hepatic uptake of pullulan in rats. J. Control. Release.

[154] Jones C.H., Chen M., Gollakota A., Ravikrishnan A., Zhang G., Lin S., Tan M., Cheng C., Lin H., Pfeifer B.A. (2015). Structure-Function Assessment of Mannosylated Poly(beta-amino esters) upon Targeted Antigen Presenting Cell Gene Delivery. Biomacromolecules.

[155] Liu Z.H., Zhang Z.Y., Zhou C.R., Jiao Y.P. (2010). Hydrophobic modifications of cationic polymers for gene delivery. Prog. Polym. Sci..

[156] Vaidyanathan S., Anderson K.B., Merzel R.L., Jacobovitz B., Kaushik M.P., Kelly C.N., van Dongen M.A., Dougherty C.A., Orr B.G., Banaszak Holl M.M. (2015). Quantitative Measurement of Cationic Polymer Vector and Polymer-pDNA Polyplex Intercalation into the Cell Plasma Membrane. ACS Nano.

