# Supplementary material for: Cationic Polymers as Transfection Reagents for Nucleic Acid Delivery
Source: Pharmaceutics. 2023 May 15;15(5):1502. doi: 10.3390/pharmaceutics15051502 (PMC10223806; doi:10.3390/pharmaceutics15051502)
Supplement: Supplementary file 1 [file pharmaceutics-15-01502-s001.zip › pharmaceutics-2342876-supplementary.pdf]

Supporting Information

Cationic polymers as transfection reagents for nucleic acid delivery

Xiaomeng Cai[1], Rui Dou[1], Chen Guo[1], Jiaruo Tang[1], Xiajuan Li[2], Jun Chen[1,*], Jiayu Zhang[1,*]

1 CAS Key Laboratory for Biomedical Effects of Nanomaterials and Nanosafety, Multi-disciplinary Research Division, Institute of High Energy Physics and University of Chinese Academy of Sciences (UCAS), Chinese Academy of Sciences (CAS), Beijing 100049, China

2 Beijing Institute of Genomics, Chinese Academy of Sciences (CAS), China National Center for Bioinformation, Beijing 100101, China

* Correspondence: chenjun@ihep.ac.cn (J.C.); zhangjy86@ihep.ac.cn (J.Z.)

Table S1. Chemical structures of different cationic polymers.

**DEAE-dextran**	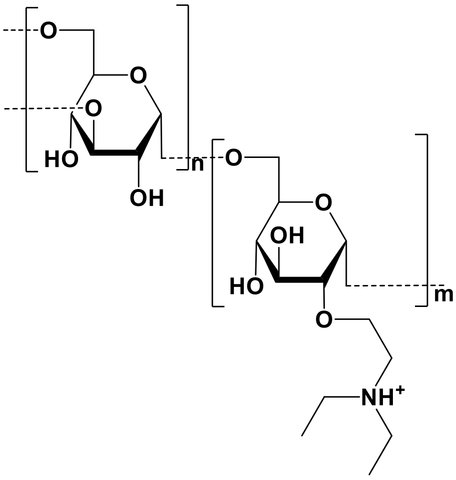
**Poly-amino acid**	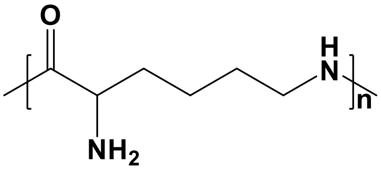
**PAMAM**	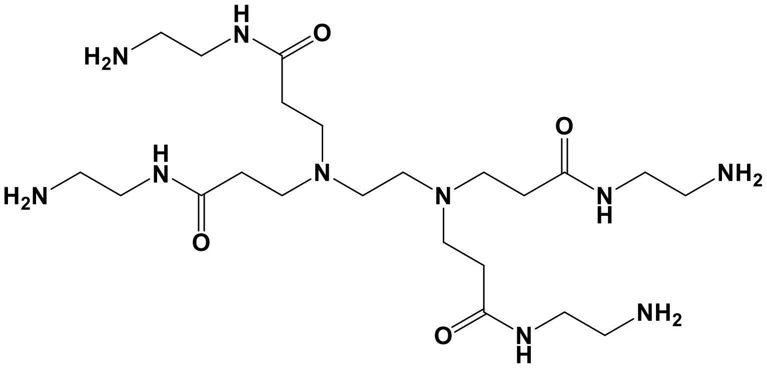
**PPI**	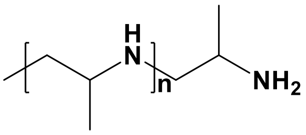
**PEI**	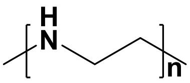 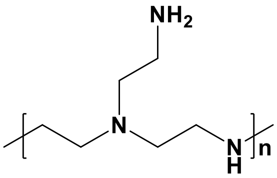
**PDMAEMA**	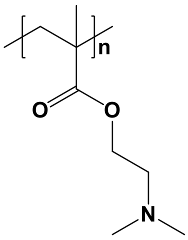

**PBAE**	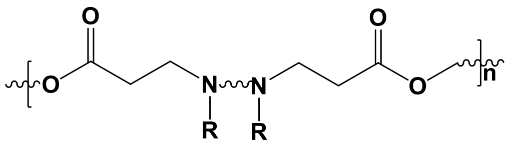
**PHP**	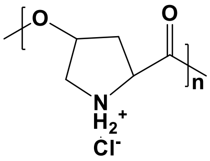
**PAGA**	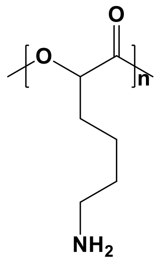
**PVL**	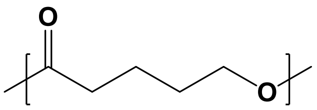
**Aminated PAHA**	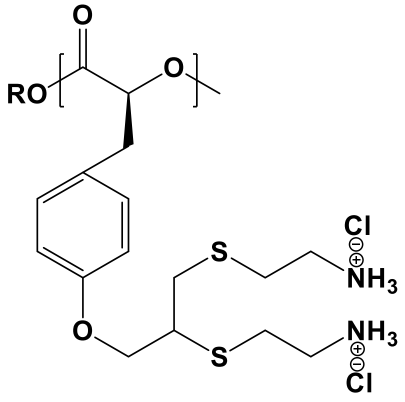
**PPE**	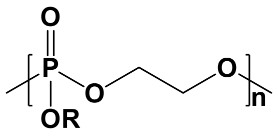
**Polylactide**	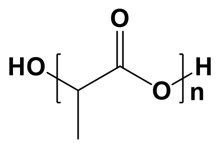
**Poly carbonate**	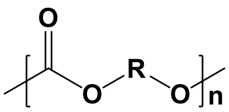
**Polyurethane**	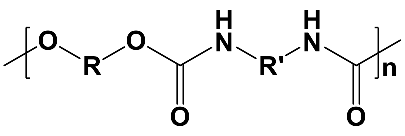
**Histone**	**N/A**

**Gelatin**	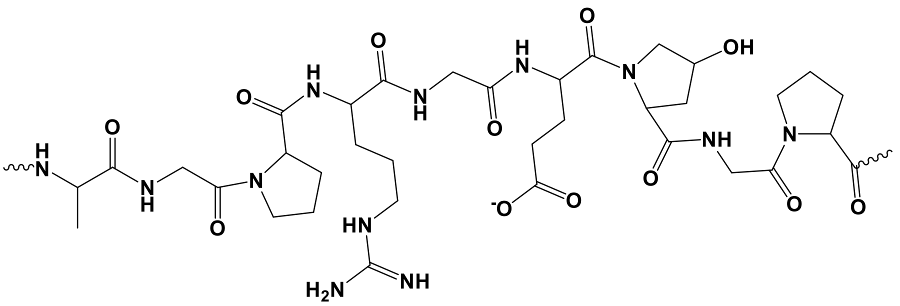
**Protamine**	**N/A**
**Cyclodextrins**	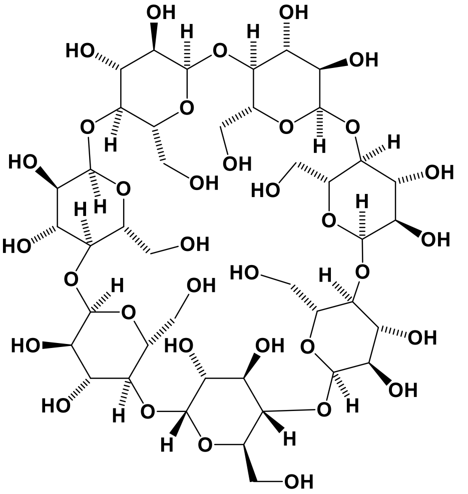
**Chitosan**	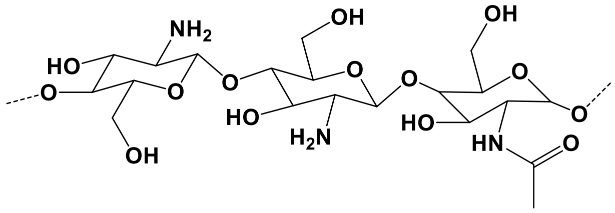

Table S2. Clinical trials with cationic polymers.

Cationic polymers	Condition	Nuclei acids	Number	State
PEI	Relapsed Neuroblastoma	DNA	NCT04049864	Recruiting
	Advanced Solid Tumors	MK-4621	NCT03739138	Terminated
	Pancreatic Adenocarcinoma	DNA	NCT01274455	Completed
	Cutaneous Squamous Cell Carcinoma<br>Basal Cell Carcinoma	DNA	NCT04925713	Completed
	Merkel Cell Carcinoma<br>Cutaneous Squamous Cell Carcinoma<br>Non-Melanoma Skin Cancers	DNA	NCT04160065	Recruiting
	Cutaneous Melanoma, Stage III<br>Cutaneous Melanoma, Stage IV<br>Merkel Cell Carcinoma<br>Cutaneous Squamous Cell Carcinoma	DNA	NCT04853602	Available
	Pancreatic Adenocarcinoma	DNA	NCT02806687	Completed
Protamine	Malignant Melanoma	mRNA	NCT00204607	Completed
	Prostate Cancer	mRNA	NCT01817738	Terminated
	Rabies	mRNA	NCT02241135	Completed
	Non-Small Cell Lung Cancer	mRNA	NCT00923312	Completed
	Non-Small Cell Lung Carcinoma	mRNA	NCT01915524	Terminated
	Hormone-refractory prostate cancer	mRNA	EudraCT-2008-003967-37	Completed
